# Stage structured prey-predator model incorporating mortal peril consequential to inefficiency and habitat complexity in juvenile hunting

**DOI:** 10.1016/j.heliyon.2022.e11365

**Published:** 2022-10-31

**Authors:** Debasish Bhattacharjee, Tapasvini Roy, Santanu Acharjee, Tarini Kumar Dutta

**Affiliations:** a Department of Mathematics, Gauhati University, Assam, India; b Department of Mathematics, Assam Don Bosco University, Assam, India

**Keywords:** Stability, Hopf bifurcation, Limit cycle, Population dynamics

## Abstract

Dynamic exploration for a predator-prey biosystem of two species with ratio-dependent functional response together with the capability for predation by both juvenile and adult stage of the predator is carried out; coupled with incentive from all the natural documentation acquainting habitat complexity as an armour for the prey and also as a factor effectuating mortality of only the juvenile predators, attributable to their inefficiency. So as to avoid extinction of either of the species and to preserve the food chain of the ecological system, the results pertaining to the existence and stability of all the equilibrium points of the biosystem along with permanence, transcritical and Hopf bifurcation have been thoroughly studied. Excessive predation by juveniles exhibits destabilization of the biosystem through supercritical Hopf bifurcation; likewise, minimal inefficiency of juveniles and habitat complexity spawns extinction and fluctuation of each species, contrarily an abundance of them decimates the predator species. The obtained results and the dependence of the biosystem on some vital parameters are corroborated from a biological viewpoint, through numerical simulation, for implementation of the system in real-life scenario. Interestingly, an illustration reveals an intriguing limit cycle between the proposed system's trivial and axial equilibriums of the proposed system along with the co-existing periodic point for specific parametric values of inefficiency of juvenile predators.

## 1 Introduction

Prey-predator dynamics are one of the most studied synergies in population ecology. The interactions between the prey and the predator can be studied with the help of mathematical models; the first ecological mathematical model was proposed by Lotka and Volterra in the first quarter of the 20th century [1], [2], [3]. For over a century, many researchers are engaged in coming up with various alterations to the foundational Lotka-Volterra model to comply with more realistic physical scenarios. In exemplar, in prey-predator models influences of some factors viz. Allee effect [55], [56], gestation delay [28], etc. are investigated. Moreover, prey-predator models have been connected with fractional calculus, for example [57]. Amidst various prey-predator models, one such modifiable reality in the model is the risk involvement in predation, which is dependent on the type of prey species [4].

In nature, the lines dividing the hunter and the hunted are often known to be blurred. The scenarios witnessed between lizards and scorpions, lobsters and whelks are that of role reversal between prey and its predator [5], [6]. The exploration done in this very field of role-reversal leaves much to be desired. The prey, when faced with their impending demise, more often than not turn towards counter-attacking its predator. Pumas are intermittently killed as a consequence of hunting porcupines or large ungulates, by being pierced or crushed by antlers or horns or being bashed into trees, or punctured by tree limbs [7]. However, it is to be noted that prey is seldom successful in killing the adult predator by reason of them being much more equipped to kill juvenile or sub-adult predators. It has been observed that the juvenile prey that was able to elude predation, eradicated juvenile predators expeditely after maturity [9]. Hence, shielding both themselves and their offsprings from predation. Few mathematical models have been proposed taking into account the role-reversal or where predators are at risk due to the anti-predator behaviour of the prey [10], [11], [12], [13], [47].

Contrary to conventional assumption, it has been evidenced that the juveniles of the predators can hunt for themselves. Snakes, juvenile walleyes, snails, fishes, *Clinus superciliosus* are among some such predators that hunt during their juvenile period of life [14], [15], [16], [17]. During predation, juvenile predators are at mortal peril when they engage with dangerous prey due to them being inexperienced and having smaller body sizes than the adults, which is more or less identical to that of the adult prey. The role-reversal scenario between adult prey *S. celarius* and juvenile phytoseiid predator can be taken as an example. It is recognized that immature phytoseiid predator may feed on adult spider mites; also the other way around it is divulged that the immature stages of the phytoseiid predator are sometimes killed by the prey spider mites, *S. celarius* [8]. It is interesting to see that adults of the predator were not harmed and could kill and feed on spider mite females and males. Moreover, researchers [27], [28], [29] studied this particular aspect of swapping of roles between juvenile predator and the prey (i.e., during predation by juveniles, either one of the prey or the juvenile may be killed) while the adult predator can successfully kill the prey without any negative ramifications.

Another scenario where prey can be termed as “dangerous prey” is diseased prey. Infectious disease is known to play an important role in nature. Many research works have been done on the theory and application of epidemiology modelling to the predator-prey population after the work of Anderson and May [18], Hadeler and Freedman [19], Chattopadhyay and Arino [20]. Infectious disease crossing the species barrier and becoming fatal for the other species has been extensively studied. Several studies revealed that the infected prey can be slain more effortlessly compared to that of non-infected prey and that the predator too would come to be infected after the consumption of these prey. Thus, the infected prey can be termed as ‘dangerous’ for the predators. The less-experienced predator would plausibly pursue the infected prey. But, we shall not foray into this particular road for our paper.

In this paper, we concentrate on assembling a biosystem where both juvenile and matured predators have the ability to hunt but only matured predators are competent enough to be immune towards prey's efforts to thwart them. To the best of our knowledge, only the authors of [27], [28], [29] have probed into this aspect, i.e., predation by juvenile-predators and its negative repercussions, through mathematical modelling (Holling type 1 functional response with and without delay is taken in these papers); but the prey taking the aid of their habitat for its anti-predator behaviour has not yet been reckoned with mathematical modelling. Envisioning the scenario where only the juveniles are at mortal peril owing to their inefficiency and prey's anti-predator deportment, which is their habitat, bereft of which the preys are not by any manner of means baneful, we study the scenario implicitly with the help of ratio-dependent functional response (the functional response that includes the rate of encountering and capturing prey since successful predation is a function of the combined probabilities of encountering and capturing prey, which are influenced by both prey's behaviour and characteristics of the terrain [39]). Prey's unique way of anti-predator behaviour, and the consideration of ratio-dependent functional response makes this endeavour quite disparate, especially from [28], [29], which fall closely to the research interest of this paper, and also from [27], [52], which study the same model as [29] but with Caputo-Fabrizio derivative and stochastic differential equation respectively; and obviously from all the papers cited above, associated with modelling role-reversal scenario between the predators and its prey mathematically.

Most of the researches advocate the concept that structurally complex habitat often impedes the pursuance of predator's forage, thereby enhancing survivorship of the prey. For example, a high-speed pursuit in an undulating landscape puts the predator at a risk of fall, rendering dislocation or breakage of limbs. Presence of dense thickets or thorny vegetation in any terrain is synonym to physical threats, inflicting debilitating injuries, particularly to eyes [4]. These injuries may act as a catalyst for causing demise of the predators. But alteration in foraging competence in the midst of development of anatomy, in different habitats within the population of the same species, has been documented in perch, stickleback, and bluegill sunfish [21], [22]. Therefore, it cannot be denied that the extent of fruition of prey surviving through complex habitat (can also be termed as refuge) is dependent upon the predator individual.

Prey females of *S. nanjingensis* have been documented to often use their nests made of silk web with the intention of locking out the immature predators (*T. bambusae*), where they subsequently died of starvation. Nonetheless, the adult predator could not be detained from invading their nests [23].

Bats, such as golden-tipped bats, *Myotis emarginatus* and the likes are known as spider-specialists as more than seventy-five percent of their diets consist of web-building spiders. There are instances of bats getting ensnared in the web of spiders and dying of exhaustion, starvation, dehydration, and/or hyperthermia (the spider may or may not directly kill/eat them); it is espied that some of the captured bats were juveniles and sub-adults, the large (adult) bats being capable of flying right through or avoid the web. Large orb weaving spiders, such as *Nephila* spp., are sometimes known to feed on bats entangled on their webs and on contrary golden tipped bats feed primarily on the orb weavers.

Also, bats that feed on insects ensnared in webs, while hovering in front of them may sometimes get entangled after inadvertently bumping into the web [24].

Recently, a scenario of snakes and spiders, which represents our model, was chronicled by Nyffeler, and Gibbons [40]. Numerous species of snakes, such as the rough green snake (*Opheodrys aestivus*) partake heavily on black widow spiders, large orb-weaving spiders and the like. The diets of some of these spiders consist of snakes caught in the web. Approaching the webs of the snake-eating spiders with the intent of capturing its inhabitants is a risky endeavour, specifically for the juvenile snakes as owing to their comparatively smaller size than that of the adults, they can get entangled irreversibly in the strong sticky webs [40].

Long-distance migration, one of the most formidable ventures undertaken by birds, has attracted a lot of attention due to its high ecological importance. In recent times, several works have been done on the migration of juvenile birds concerning their mortality, wind favourability, navigation, etc. [41], [42], [43], [44]. Juvenile birds with no prior experience, rely completely on their innate navigation potentialities during this task. According to documentation birds with inferior capabilities stipulate an increased number of stopover sites for food, recuperation and other essentials. Supplementarily birds in poorer conditions undertake more risks while pursuing a prey. This potential risk of injury or death may be in the form of unfamiliar prey and/or unfamiliar surroundings of the prey [45].

Another picture that can be painted with our proposed model is that of between the top-predator, mesopredator, and the prey. Both the predators are territorial and the top-predator is a specialist predator while mesopredator is a generalist one. During ontogenesis, juveniles of the top-predator are not invulnerable to mesopredators; hence when they step into mesopredators' territory to forage for its prey, they may get killed, while the adults have no such constraints as they can overpower the mesopredators. For example, wolves and coyotes can be taken as the top and intermediate predators, respectively. Wolves feed specifically on white-tailed deer; coyotes, as generalist omnivore, have a much more variable diet. Coyotes are known to attack and harass wolves in neutral or their own territories (both of their territories are sometimes known to overlap) [25], [26]. For our model, we can take wolves as our predator and white-tailed deers as our prey.

All these natural evidences catalogued in literature encouraged us to discuss the mathematical model with stage structure in the predator, to reflect upon the following questions:

(i) How do inefficiency of the juvenile predators and the habitat complexity, owing to which only juvenile predators are racked with mortal risks have ascendancy over the whole biosystem?

(ii) Can we connect Allee effect, gestation delay, etc. in prey-predator models where factors like juvenile predators are involved?

In this paper, we will confine only to the first question. The construction of the mathematical model is discussed in section 2. In section 3, non-negativity and boundedness of the solution have been investigated. In section 4, the equilibrium points are discussed along with the local and global stabilities of the system at these points. In section 5, uniform persistence is analyzed. In section 6, transcritical and Hopf bifurcation is studied. Finally, numerical simulations are presented, followed by the conclusion.

## 2 Mathematical construction of the biosystem

For the construction of the biosystem concerning two interacting species, the prey and the predator, the prey is taken to have a logistic growth rate in the absence of the predator, owing to limited resources in nature. Let X(T) and Y(T) be biomass densities of the prey and the predator, respectively, *r* is the intrinsic growth rate, and *K* the carrying capacity of the environment. Thus, the logistic equation is given below:

$$\overset{˙}{X}=Xr(1-\frac{X}{K}),\;X(0)>0.$$

Functional response is a key element in the mathematical model governing the predator-prey interaction. All the functional responses can be grouped into three different types of functional responses:

1. prey dependent,
2. predator dependent,
3. multi-species dependent.

In prey-dependent, the functional response is affected only by prey. Holling I, II, III, IV, Ivlev type and Rosenzweig type are such types of functional responses. In the simplest form, a prey-dependent predator-prey model is given as:

$$\overset{˙}{X}=Xf(X/K)-Yg(X),\overset{˙}{Y}=\mu_{1}Yg(X)-DY,$$

with X(0)>0, Y(0)>0.

Here, *D* is the natural death rate of the predators, $\mu_{1}$ is the conversion rate of killed prey into juvenile predator.

Beddington–DeAngelis type, Crowley–Martin type, Hassell–Varley type are the examples of predator-dependent functional responses. Such types of functional responses are affected by both predator and prey populations. Generally, they are of the following forms:

$$\overset{˙}{X}=Xf(X/K)-Yh(X,Y),\overset{˙}{Y}=\mu_{1}Yh(X,Y)-DY,$$

with X(0)>0, Y(0)>0.

In multi-species dependent functional response, not just the focal prey and predator species, but other species and subsidiary elements that may be of some consequences to the functional response are incorporated. It is generally used when three or more species are involved. Modelling multi-species systems is a complex task, and in these models, the functional response has a particularly important role to play [34].

In our model, we employ ratio-dependent functional response. This is a particular type of predator dependent functional response, which relies on the ratio of prey population size to the predator population size, rather than the definite number of either of the species. This functional response is a finer fit for our model as search and conquer rate is supposed to be at a substantial position to affect the interaction between the predator and the prey. Many biologists accredit ratio-dependent functional response to be more propitious in depicting real-life scenarios [30], [31], [32]. The general form of ratio-dependent functional response is as follows:

$$\overset{˙}{X}=Xf(X/K)-p(Y/X),\overset{˙}{Y}=\mu_{1}p(Y/X)-DY,$$

with X(0)>0, Y(0)>0.

Partitioning the predator species into juvenile and matured stages, $Y_{j}$ and, $Y_{m}$ are their biomass densities, respectively. Both juvenile and matured stages possess the ability of predation and follow the ratio dependent functional responses, $\phi_{1}(\frac{Y_{j}}{X},\frac{Y_{m}}{X})$ and, $\phi_{2}(\frac{Y_{j}}{X},\frac{Y_{m}}{X})$, respectively. During ontogenesis, the juvenile reproductive system does not fully develop, and hence only the adults reproduce. Predation by adults corresponds to continuation of the species, i.e., reproduction, thereby enhancing the biomass density of their younger ones. While successful predation by juveniles conforms to their own survival and has no impact on the changes in the biomass density.

$$\overset{˙}{X}=Xr(1-X/K)-Y_{j}\phi_{1}(\frac{Y_{j}}{X},\frac{Y_{m}}{X})-Y_{m}\phi_{2}(\frac{Y_{j}}{X},\frac{Y_{m}}{X}),\overset{˙}{Y_{j}}=\mu_{1}\phi_{2}(\frac{Y_{j}}{X},\frac{Y_{m}}{X})Y_{m}-CY_{j}-D_{1}Y_{J},\overset{˙}{Y_{m}}=CY_{j}-D_{2}Y_{m},$$

with X(0)>0, $Y_{j}(0)>0$, $Y_{m}(0)>0$.

Here, *C* is the maturation rate of juvenile predator, $D_{1}$ and $D_{2}$ are natural death rates of juvenile and matured predator respectively.

The success of juvenile predators in killing the prey is substantially interconnected to the prey being outside of their habitats or the juvenile predator staying out of the mesopredator's territory (for simplification, we shall go by habitat complexity). Assimilating habitat complexity leaves *X(1-n)* of the prey that are available for juvenile predator to hunt. The functional response of juveniles is transformed to $\phi_{1}(\frac{Y_{j}}{X(1-n)},\frac{Y_{m}}{X(1-n)})$, where n∈[0,1] is the habitat complexity. In addition to the lack of robustness, juvenile predators have scant knowledge and experience in predation, making them inefficient. The mortal peril due to this inefficiency has been factored into our biosystem as $\phi_{3}(\frac{Y_{j}}{X},\frac{Y_{m}}{X})$. The negative repercussions of predation by juveniles when coupled with the habitat complexity are remoulded to $n\phi_{3}(\frac{Y_{j}}{X},\frac{Y_{m}}{X})$. Habitat complexity is omitted in the functional response, since it doesn't hinder the juvenile predator from getting killed, but rather aids, and hence the multiplication. The root cause that warrants the deaths of juvenile predators is not the prey themselves but their habitat complexity, in the absence of which (n=0), the juveniles would be at complete liberty to hunt each and every prey individual without experiencing any ramifications. Also, in the course of events where juveniles are fully efficient (B=0), the presence of habitat complexity would not result in their deaths, a lot like the scenario with the matured predators.

$$\phi_{1}=A_{1}/(m(\frac{Y_{j}}{X(1-n)}+\frac{Y_{m}}{X(1-n)})+1),\phi_{2}=A_{2}/(m(\frac{Y_{j}}{X}+\frac{Y_{m}}{X})+1),\phi_{3}=B/(m(\frac{Y_{j}}{X}+\frac{Y_{m}}{X})+1),$$

where *m* is the average search and conquer rate of juvenile and matured predator, $A_{1}$, and $A_{2}$ being the predation rates of juvenile and matured predator respectively, and *B* is the inefficiency rate of juvenile predators.

So, our biosystem is:

(1) $$\begin{array}{cc} \frac{dX}{dT} & =rX(1-\frac{X}{K})-\frac{A_{1}(1-n)XY_{j}}{\left(m(Y_{j}+Y_{m})+X(1-n)\right)}-\frac{A_{2}XY_{m}}{\left(m(Y_{j}+Y_{m})+X\right)},\\ \frac{dY_{j}}{dT} & =\frac{\mu_{1}A_{2}XY_{m}}{\left(m(Y_{j}+Y_{m})+X\right)}-\frac{BnXY_{j}}{\left(m(Y_{j}+Y_{m})+X\right)}-CY_{j}-D_{1}Y_{j},\\ \frac{dY_{m}}{dT} & =CY_{j}-D_{2}Y_{m}, \end{array}$$

with initial conditions

$$X(0)>0,\;Y_{j}(0)>0,\;Y_{m}(0)>0.$$

Now, simplifying the biosystem by reducing the number of parameters by taking t=rT, x=X/K, $y=Y_{j}/K$, $z=Y_{m}/K$, $A_{3}=A_{2}\mu_{1}$, the equations of (1) are now transformed to:

(2) $$\frac{dx}{dt}=x(1-x)-\frac{a_{1}(1-n)xy}{\left(m(y+z)+(1-n)x\right)}-\frac{a_{2}xz}{\left(m(y+z)+x\right)},\frac{dy}{dt}=\frac{a_{3}xz}{\left(m(y+z)+x\right)}-\frac{bnxy}{\left(m(y+z)+x\right)}-cy-d_{1}y,\frac{dz}{dt}=cy-d_{2}z,$$

with initial conditions:

(3) $$x(0)=x_{0}>0,\;y(0)=y_{0}>0,\;z(0)=z_{0}>0,$$

where, $a_{1}=A_{1}/r$, $a_{2}=A_{2}/r$, $a_{3}=A_{3}/r$, b=B/r, $c=C_{1}/r$, $d_{1}=D_{1}/r$, and $d_{2}=D_{2}/r$.

## 3 Non-negativity and boundedness

From biological point of view, the prey having logical growth rate, cannot grow exponentially and due to the reliance of the predator population on the prey population, they, too, are confronted with an identical scenario. The following theorems attest to this very fact of the biosystem (2) being restrained within a particular region in the positive octant, numerically.

Theorem 1 *Every single one of the solutions of the biosystem* (2) *with initial conditions* (3) *are non-negative.*

Theorem 2 *The solutions of the biosystem* (2) *along with initial condition* (3) *are uniformly bounded in* $\left\{(x,y,z)\in R_{+}^{3}:p=\frac{a_{3}{\left(1+d_{3}\right)}^{2}}{4d_{3}}-\tau ,\;\text{for any}\;\tau >0\right\}$ *, the values of p,* $d_{3}$ *are* $a_{3}x+a_{2}(y+z)$ *and* $min\{d_{1},d_{2}\}$ *respectively.*

Refer to Appendix A and Appendix B for the proofs.

## 4 The equilibrium points and their stability

The ecological system experiences three types of equilibrium, which are bionomically realizable. One of them is the obvious vanishing equilibrium $E_{1}(0,0,0)$. When the preys are not disrupted by the predators, they cherish in the ecological system to the full potential of the system as depicted by the model. Therefore, the long-term state of the ecological system would be the equilibrium point $E_{2}(1,0,0)$. At both the stages, predators are specialist predators and hence, the existence of other types of axial equilibrium is inconceivable. One of the best scenarios of an ecological system is the existence and stability of the co-extant equilibrium $E_{3}(x^{⁎},y^{⁎},z^{⁎})$. In this section, the requirements for all the equilibrium points to be locally and globally stable have been investigated.

1. **The Vanishing Equilibrium point**$E_{1}(0,0,0)$
  The trivial solution (0,0,0) is a saddle point. The stable and unstable manifolds have been investigated along the directions of different axes and planes. The trajectories advancing through either y-axis, z-axis or yz-plane end up at (0,0,0).
  Refer to Appendix C for the proof.
2. **The Axial Equilibrium or the predator free equilibrium point**$E_{2}(1,0,0)$
  Theorem 3 *The system around the axial point*(1,0,0)*is locally asymptotically stable if*
  $$0<a_{3}<\frac{\left(cd_{2}+d_{1}d_{2}+bd_{2}n\right)}{c}.$$
  Theorem 4 *The sufficient condition for the axial equilibrium point of the biosystem*(2)*along with the initial values to be globally asymptotically stable is*$a_{3}z-bnxy<0$*and*$a_{1}a_{3}(1-n)(1-x)<a_{2}m(d_{1}y+d_{2}z)$*.*
  Refer to Appendix D and Appendix E for the proofs.
3. **The Co-extant equilibrium point**$E_{3}(x^{⁎},y^{⁎},z^{⁎})$
  The equilibrium point $\left(x^{⁎},y^{⁎},z^{⁎}\right)$ is given by
  $$x^{⁎}=\frac{u}{(c+d_{2})m(a_{3}c-bd_{2}n)v},y^{⁎}=\frac{c(a_{3}c-d_{2}(c+d_{1}+bn))u}{m^{2}v(c+d_{1}){\left(c+d_{2}\right)}^{2}(a_{3}c-bd_{2}n)},z^{⁎}=\frac{cu(a_{3}c-d_{2}(bn+c+d_{1}))}{d_{2}m^{2}v(c+d_{1}){\left(c+d_{2}\right)}^{2}(a_{3}c-bd_{2}n)},$$
  where,
  $u=a_{2}c[a_{3}c-(b+c+d_{1})d_{2}n][a_{3}c-d_{2}(c+d_{1}+bn)]-(a_{3}c-bd_{2}n)[(c+d_{2})m[a_{3}c-(b+c+d_{1})d-2n]-a_{1}d_{2}(-1+n)(-a_{3}c+d-2(c+d_{1}+bn))]$,
  and $v=-a_{3}c+(b+c+d_{1})d_{2}n$.
  Theorem 5 *The necessary and sufficient conditions for the co-extant equilibrium point to exist are*
  *(i)* $\left(a_{3}c)>d_{2}(c+d_{1}+bn\right)$*,*
  *(ii)* $a_{2}c[a_{3}c-d_{2}(bn+c+d_{1})]<m(c+d_{2})(a_{3}c-bd_{2}n)$*,*
  *(iii)* $0<a_{1}<[\{a_{3}c-d_{2}n(b+c+d_{1})\}\{a_{2}c(a_{3}c-d_{2}(bn+c+d_{1}))-m(c+d_{2})(a_{3}c-bd_{2}n)\}]/[d_{2}(n-1)(bd_{2}n-a_{3}c)(d_{2}(bn+c+d_{1})-a_{3}c)]$*.*
  The proof of the above theorem is obvious and hence, we omitted it.
  Existence of co-extant equilibrium point necessitates instability of predator free equilibrium point, as the first condition given above is in contradiction to Theorem 3.
  Theorem 6 *The equilibrium point*$E_{3}(x^{⁎},y^{⁎},z^{⁎})$*is locally asymptotically stable if*$\chi_{1}>0$*,*$\chi_{3}>0$*,*$\chi_{4}>0$*, where the values of*$\chi_{1},\chi_{3},\chi_{4}$*are given within the proof.*
  One can see Appendix F.
  Theorem 7 *The system of equations*(2)*will be globally asymptotically stable around co-extant equilibrium point*$E_{3}(x^{⁎},y^{⁎},z^{⁎})$*, if*ϑ<0*where the value of ϑ is given within the proof.*
  The global stability of the co-extant equilibrium point has been discussed (in Appendix G) with the help of geometric approach given by Li and Muldowney [36] with a parallel technique as given in [12].

## 5 Perseverance

A system of equations can be asserted to persevere for eons if there is an existence of a compact region $E\subset {R^{3}}_{+}$ with the property that all the solutions of the system with its initial condition eventually enter and reside inside E.

For the perseverance of the biosystem (2) with initial conditions (3), we need positive constants 0<α≤β [53], [54] such that

(4) $$max\{\lim_{t\to \infty }sup\;x(t),\lim_{t\to \infty }sup\;y(t),\lim_{t\to \infty }sup\;z(t)\}\leq \beta$$

(5) $$min\{\lim_{t\to \infty }inf\;x(t),\lim_{t\to \infty }inf\;y(t),\lim_{t\to \infty }inf\;z(t)\}\geq \alpha$$

Equation (4) is already proven through boundedness of the biosystem in the Theorem 2. So, only equation (5) is proven in Appendix H.

Theorem 8 *The biosystem* (2) *perseveres if* $a_{3}c>d_{2}(bn+c+d_{1})$ *and* $m>a_{1}(1-n)+a_{2}$ *.*

## 6 Bifurcation

### 6.1 Hopf bifurcation

**Analysis of Hopf bifurcation**

We orient towards constituting the criteria for Hopf bifurcation around the co-extant equilibrium point $E_{3}(x^{⁎},y^{⁎},z^{⁎})$ with respect to a parameter. Suppose $n_{h}$ is the point of bifurcation for the parameter *n* (habitat complexity). The necessary and sufficient condition for $n_{h}$ to be Hopf bifurcation point is:

1. $\chi_{i}(n_{h})>0$ for i=1,2,3,
2. $\chi_{1}(n_{h})\chi_{2}(n_{h})=\chi_{3}(n_{h})$,
3. ${\left[\frac{d}{dn}(\chi_{1}\chi_{2}-\chi_{3})\right]}_{n=n_{h}}\neq 0$.

**Direction and stability of bifurcating periodic solution**

Theorem 9 *The sign of* $\mu_{2}$ *clarifies the trajectory of Hopf bifurcation. The biosystem* (2) *encounters supercritical bifurcation for a positive* $\mu_{2}$ *and subcritical bifurcation when* $\mu_{2}<0$ *.* $\beta_{2}$ *indicates the stability of the bifurcating periodic solution; it is stable for negative* $\beta_{2}$ *and unstable for a positive* $\beta_{2}$ *. The period of bifurcating periodic solution escalates when* $T_{2}>0$ *and diminishes when* $T_{2}<0$ *.*

### 6.2 Transcritical bifurcation

Theorem 10 *The biosystem*(2)*is subjected to transcritical bifurcation around the predator free equilibrium point*$E_{1}$*concerning*$b=b_{t}=(a_{3}c-cd_{2}-d_{1}d_{2})/nd_{2}$*.*

It is to be noted that we can also take other parameters as bifurcating parameter.

## 7 Numerical simulation

In this section, we perform rigorous numerical simulations taking the assistance of MATLAB software using odes45, MATCONT [35] and MATHEMATICA software. The intent for the numerical simulation is to both authenticate the theoretical results developed in the previous sections as well as explicate the rich dynamical scenario under the ascendancy of different parameters. Some hypothetical biologically feasible values of parameters have been considered as shown in the following table:

**Table 1:** Values of parameters.

Physical meaning	Parameter	Value
Predation rate by juvenile predator	*a*_1_	1.25
Predation rate by matured predator	*a*_2_	1.4
Conversion rate of juveniles	*a*_3_	1.8
Inefficiency rate of predation by juveniles	*b*	1.015
Search and conquer rate	*m*	0.95
Mortality risk of juvenile due to habitat complexity	*n*	0.5
Transformation rate to adult	*c*	0.09
Natural death rate of juvenile	*d*_1_	0.2
Natural death rate of adult	*d*_2_	0.1

The parametric values would change when given otherwise.

### 7.1 Numerical verification of the equilibrium points

We consider b=1.5, n=0.9 and rest of the parameters from Table 1. Different initial points as shown in the Fig. 2 containing co-extant population leads to predator-free equilibrium through different trajectories, which confirms the presence of global stability of the axial equilibrium. It stands to reason that presence of global stability implies local stability. The values of the parameters satisfy the local stability condition of the predator-free equilibrium, since we have $\left(\frac{d_{2}(c+d_{1}+bn)}{c}=1.8222\right)>(a_{3}=1.8)$, thereby validating the numerical simulation. Furthermore, the condition of global stability of axial equilibrium point (Theorem 4) being a sufficient one, other trajectories, not satisfying the condition also leads to the point (1,0,0).

However, when the values of all the parameters are considered as given in the table (i.e., values of *b* and *n* are decreased when compared to Fig. 2), $E_{1}$ loses its stability and the co-extant equilibrium point $E_{3}(0.189539,0.297824,0.268042)$ is found to be existent attributable to $0<\frac{\left(cd_{2}+d_{1}d_{2}+bd_{2}n\right)}{c}<a_{3}$ (Theorem 3 and Theorem 5, respectively). The local stability, indicated by the Routh-Hurwitz criteria developed in Theorem 6 is satisfied as we have, $\xi_{1}=0.646$, $\xi_{3}=0.004064$, $\xi_{4}=0.008969$, i.e. $\xi_{1},\xi_{3},\xi_{4}>0$. Figs. 3(a-d) attest to the numerical reasoning, as all the three populations initializing at different points advance towards their respective populace according to $E_{3}$. It stands to reason that varying the rates of inefficiency and habitat complexity can change the behaviour of the biosystem concerning the equilibrium points.

However, for this set of values, neither the conditions of global stability nor perseverance is obeyed numerically. For the verification of the condition of global stability of co-extant equilibrium point (Theorem 7), we have taken another set of parameter values, $a_{1}=0.75$, b=0.7, m=1.95, n=0.28 and the rest, as given in the table. For these values, we acquire $\xi_{1}=1.0927$, $\xi_{3}=0.014187$, $\xi_{4}=0.32469$ along with ϑ=−0.0027871<0, which is the condition of global stability. The graphical illustration in Fig. 4 validates the existence of a neighbourhood satisfying global stability. Condition of perseverance (Theorem 8) is also satisfied at these parameters, because of $\left[(a_{1}(1-n)+a_{2}=1.94)<(m=1.95)\right]$ and $\left[\left(\frac{d_{2}(c+bn+d_{1})}{c}=0.54\right)<(a_{3}=1.8)\right]$. So theoretically, the initial co-extant population will co-exist perpetually. Stability of the co-existing equilibrium supports this fact as the trajectories taking different initial points stay in the co-existing equilibrium in the long run.

### 7.2 Bifurcations

Verification of the Hopf bifurcation conditions and its direction is performed prior to the exploration of the occurrence of periodic oscillations and its nature among the three populations with respect to the different bifurcation parameters. By reason of evaluation of the parameters for Hopf bifurcation and its direction, numerically, we first consider the parameter $a_{1}$, the predation rate by juveniles. The biosystem (2) undergoes Hopf bifurcation at $a_{1}=a_{1h}=1.350114$ as it satisfies the NASC condition for Hopf bifurcation i.e., $\xi_{1}=0.618917$, $\xi_{3}=0.00355832$, $\xi_{4}=0$ and $\frac{d\xi_{4}}{da_{1}}=-0.0856855(\neq 0)$ at $a_{1}=a_{1h}$.

As specified in Theorem 9, we are able to find the nature and direction of bifurcating periodic solution. From the theories with the above-mentioned set of parametric values we get,

$$g_{11}=-0.199376-0.0174382i,\;g_{02}=-0.493577-0.043236i,$$

$$g_{20}=-0.120705+0.180489i,\text{ and }g_{21}-0.929725+0.771532i.$$

Hence, we get, $C_{1}(0)=-0.0996479-0.874509i$, $\mu_{2}=0.904326$ and $\beta_{2}=-0.199296$.

Here, $\mu_{2}$ being greater than 0 attests the bifurcation to be a supercritical one and, as $\beta_{2}<0$, thus the periodic cycle is stable.

Figure 5, Figure 6, Figure 7, agreeing with the numerical calculations, show the phenomenon of bifurcations. At $a_{1}=a_{1h}=1.350114$, there is a birth of limit cycle (Figs. 6(a-b)) as the initial co-existing point follows an oscillation. The amplitude of this oscillation keeps on diminishing as the value of $a_{1}$ diminishes and the system evinces stable focus point. Figs. 5(a-b) show this fact at $a_{1}=1.32<a_{1h}$. There is a visible increase in the amplitude of oscillation at $a_{1}=1.37>a_{1h}$ (Figs. 7(a-b)), the co-extant equilibrium point becomes unstable as the parameter crosses the threshold value $a_{1}=a_{1h}$ and the system exhibits limit cycle oscillations.

Similarly, when considering the parameter of habitat complexity (*n*) as the bifurcating parameter, $n=n_{h}=0.46633256$ is found to be the point of Hopf bifurcation as we have, $\xi_{1}=0.609403$, $\xi_{3}=0.003585$, $\xi_{4}=0$, and $\frac{d\xi_{4}}{dn}{|}_{n=n_{h}}=0.24348$. Further, we have

$$g_{11}=-0.199062-0.0140613i,\;g_{02}=-0.497739-0.0377014i,$$

$$g_{20}=-0.120799+0.186913i,\;g_{21}=-0.864663+0.896075i,$$

$$\mu_{2}=0.824864\text{ and, }\beta_{2}=-0.401689.$$

With $\mu_{2}>0$ and $\beta_{2}<0$, the supercritical bifurcation occurs, this is shown in Fig. 10, where the stable periodic cycles can be seen at n=0.42 ((a) of Fig. 10).

Next, the parameter $a_{3}$ is examined. This parameter is pertinent to growth factor of the juvenile predator. It harbours the capacity to cause both transcritical and Hopf bifurcation (see section 6). Considering the given set of parametric values from Table 1, at $a_{3}=0.8861111$ there is reciprocity of stability between axial equilibrium point and co-extant equilibrium point due to transcritical bifurcation. At $a_{3}=a_{3h}=2.0326485$, there is a supercritical Hopf bifurcation, the first Lyapunov coefficient being $-3.601898e^{-02}$, exchanging the stability from the co-existing equilibrium to a stable limit cycle being born around the said equilibrium point, which becomes unstable. The biosystem is observable to maintain a stable steady state corresponding to predator-free equilibrium in the domain $0\leq a_{3}<0.886111$. This circumstance is succeeded by a stable steady state where each population attains its optimum number in the domain $0.886111\leq a_{3}<a_{3h}$. As the parametric value of $a_{3}$ is continuously increased after the critical value $a_{3h}$, the amplitude of fluctuation in all the populations too would increase, Figs. 8(a-c) visualize this very scenario, where the solid line denotes the stable co-extant equilibrium with respect to the variation in conversion rate of juveniles ($a_{3}$), up to $a_{3h}$, after which point the amplitude of oscillation is descried to be escalating.

It can be therefore concluded for an excessive conversion rate, the populations of both the predator and prey species progress towards extinction due to the fact prey population could not accommodate the dietary requirements of the growing population of predators, at the same time, low conversion rate makes it really arduous for the predator species to survive resulting in their extinction.

Similarly, for the searching rate of both the predator, taken as average (*m*), Figs. 9(a-c) visualize the backward bifurcation with respect to *m*. The point of bifurcation being m=0.9291448. For m>0.9291448, the system follows a stable trajectory for co-extant equilibrium point, denoted by a straight horizontal line in Fig. 9. The first Lyapunov coefficient at m=0.9291448 is found to be $-1.110749e^{-01}<0$, hence a supercritical Hopf bifurcation. As *m* decreases, the fluctuations in prey, juvenile predator and matured predator populations increase. Now, if the parametric value is further decreased, the limit cycle that arises too would cease to exist after a certain value of the parameter. The co-extant equilibrium point being unstable, the trajectory goes to the vanishing equilibrium point. That being the case, the collapse of the whole biosystem can be triggered when the predators can frequently and effortlessly kill their prey.

### 7.3 Influence of inefficiency and habitat complexity

**Figure 1:**
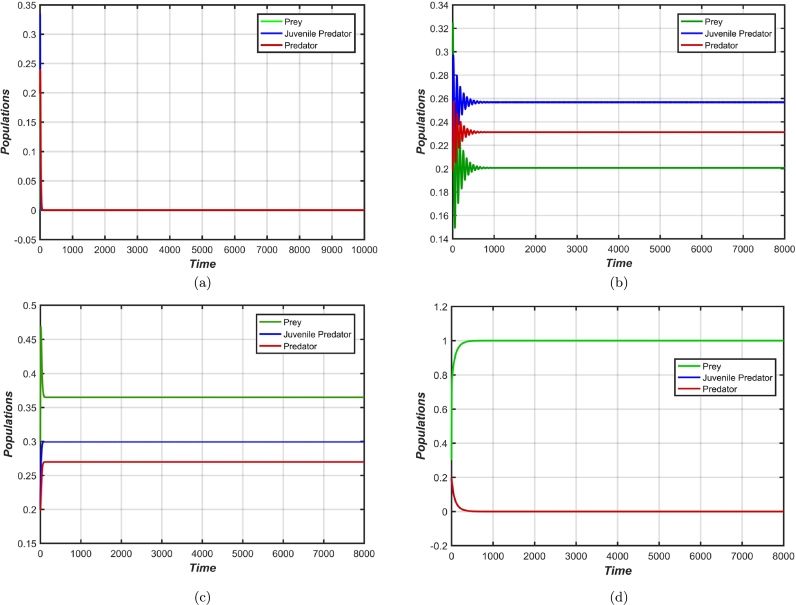
Alteration in populations dynamics with varying value of *n* (anti-predator behaviour of prey due to habitat complexity), viz: at (a) *n* = 0, (b) *n* = 0.44, (c) *n* = 0.65, and (d) *n* = 1, with other parameter values: *a*_1_ = 1.25, *b* = 1.5, *a*_2_ = 1.4, *a*_3_ = 1.8, *m* = 0.95, *c* = 0.09, *d*_1_ = 0.2 and, *d*_2_ = 0.1.

To comprehend the significance of habitat complexity (*n*) in determining the behaviour of the biosystem, Figs. 1(a-d) are drawn at different set of values of *n*. Other parameters are taken as given in Table 1 except for b=1.5. For n=1, the system harbours a stable predator-free equilibrium (Fig. 1d), while for n=0, the system goes towards the vanishing equilibrium (Fig. 1a). For values greater than 0, the co-extant equilibrium point obtains its stability, i.e., for $n>n_{h1}$, where the point $n=n_{h1}=0.42286752$ accommodates a supercritical Hopf bifurcation. For $n\in (n_{h1}n_{h2})$ the co-extant equilibrium is stable (Figs. 1b and 1c), here $n_{h2}=0.886667$. In Fig. 1b, n=0.44 being near $n_{h1}$, we can see a little oscillation with diminishing amplitude in all three populations before settling into the optimum populace, while n=0.65 (Fig. 1c) being farther away from $n_{h1}$, no oscillation can be seen. The system stability is reassigned from co-extant equilibrium point to predator-free equilibrium point at $n_{h2}$ as a result of transcritical bifurcation. For values higher than $n_{h2}$, the system nestles into a stable predator-free equilibrium, as is descried in Fig. 1d.

**Figure 2:**
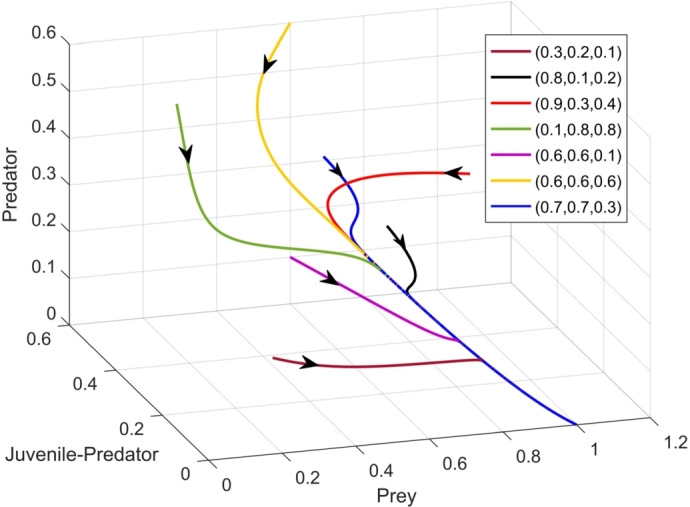
Axial equilibrium point *E*_2_(1,0,0) being globally asymptotically stable.

**Figure 3:**
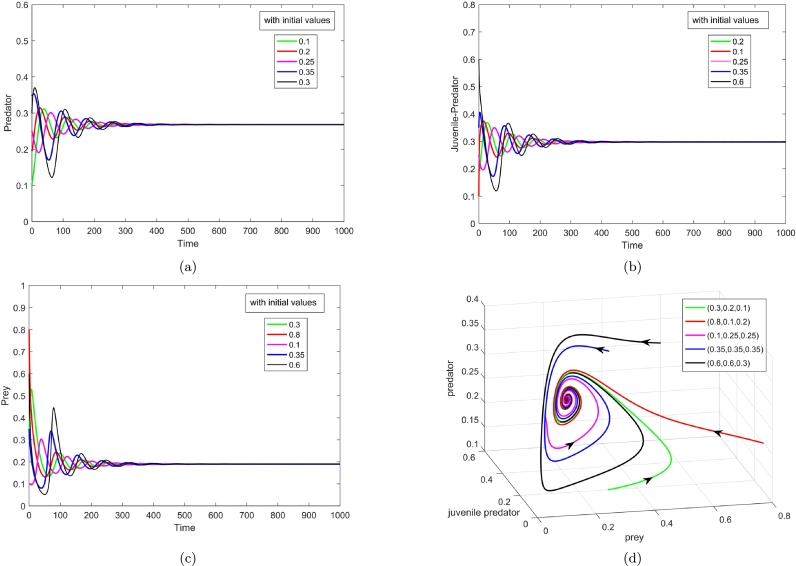
Local stability of co-extant equilibrium *E*_3_(*x*^⁎^,*y*^⁎^,*z*^⁎^), with respect to (a) predator population, (b) juvenile predator population, (c) prey population, and (d) phase portrait.

**Figure 4:**
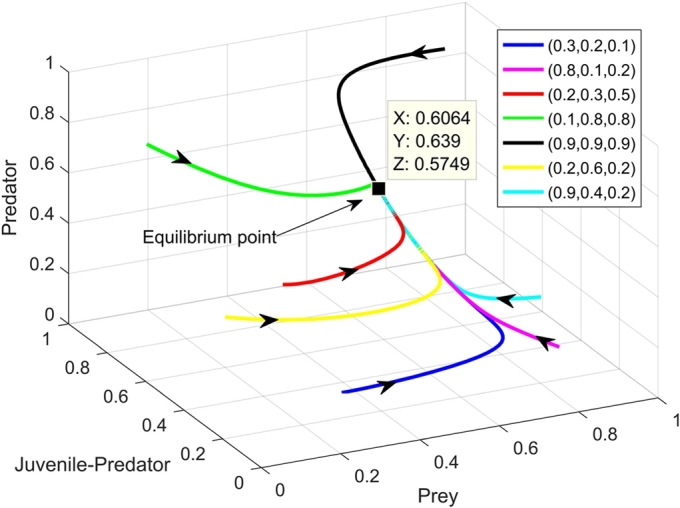
Global stability of co-extant equilibrium.

**Figure 5:**
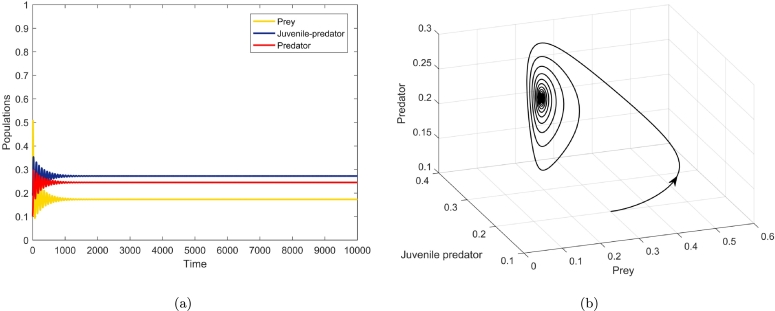
At *a*_1_ = 1.32 < *a*_1*h*_, (a) shows time series of prey, juvenile predator and predator populations, and (b) depicts phase portrait.

**Figure 6:**
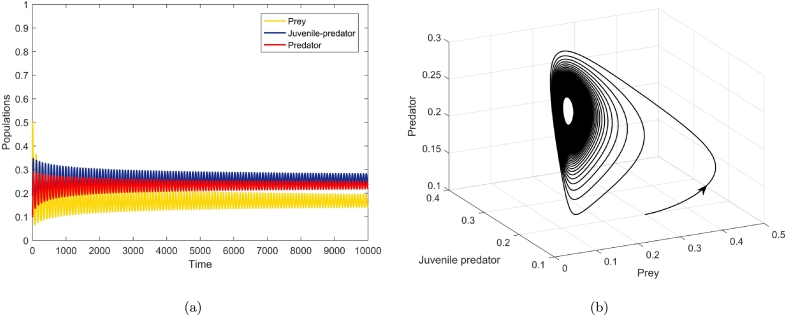
At *a*_1_ = *a*_1*h*_ = 1.350115, the solutions of the biosystem (2) exhibiting emergence of periodic oscillations through (a) time series, and (b) phase portrait around the co-extant equilibrium point.

**Figure 7:**
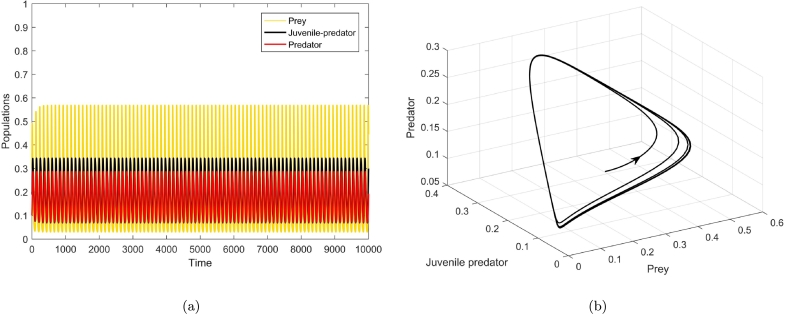
At *a*_1_ = 1.37 > *a*_1*h*_, (a) depicts time series of the three populations, and (b) depicts the phase portrait of equilibrium point *E*_3_(*x*^⁎^,*y*^⁎^,*z*^⁎^), where it is unstable but the limit cycle is stable.

**Figure 8:**
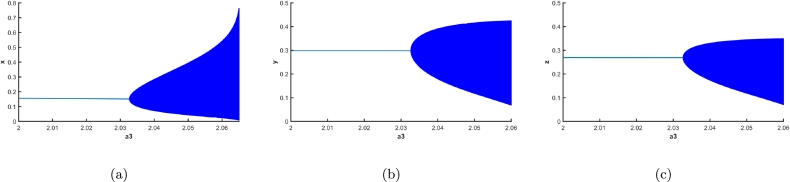
Forward bifurcation with respect to conversion rate of juveniles *a*_3_ is depicted for (a) prey population, (b) juvenile predator population and, (c) adult predator population.

**Figure 9:**
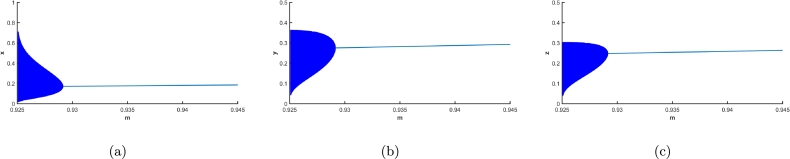
Backward bifurcation with respect to search and conquer rate *m* is depicted for (a) prey population, (b) juvenile predator population and, (c) adult predator population.

**Figure 10:**
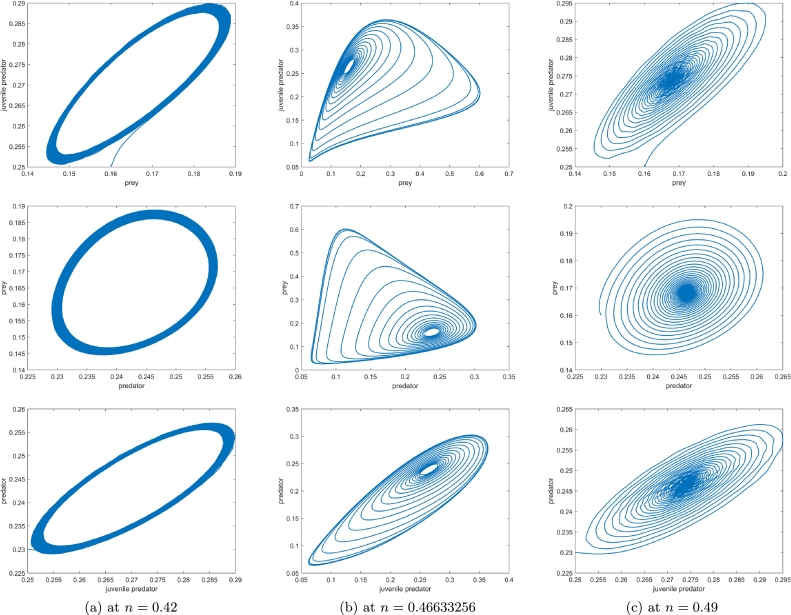
Portraying prey population, juvenile and matured predator population dynamics in a bi-dimensional space with (a) *n* < *n*_*h*_, (b) *n* = *n*_*h*_ and, (c) *n* > *n*_*h*_, where *n*_*h*_ = 0.46633256 is Hopf bifurcation point.

The Hopf bifurcation plot with respect to *n* is portrayed in Figs. 10(a-c) (with b=1.015), the bifurcating parametric value is at n=0.46633256. The initial co-existing population follows a trajectory that spirals into the co-extant equilibrium point at n=0.49 (Fig. 10c) and spirals around the said equilibrium point at n=0.46633256 (Fig. 10b). A stable limit cycle with unstable equilibrium point at its focus can be seen at n=0.42 (Fig. 10a). Biologically, with the habitat complexity too high, the survival of juvenile predators becomes really difficult leading to extinction of the whole predator species, while with less habitat complexity, the juvenile predator can easily survive and kill the prey, which would initially increase the biomass density of the predator species but would lead to collapse of the whole biosystem in the long run. Also, owing to the existence of supercritical Hopf bifurcation, populations of both the stages of predator species and the prey species not being able to conform to an explicit optimum number of individuals, are seen to be oscillating with a certain amplitude. Fig. 10a shows this phenomenon for n=0.42, the limit cycle explicates that when both the species reach the highest obtainable population, the biomasses start decreasing, bringing about the lowest obtainable populations of both species, and once that too is conceived, the biodensities commence escalating anew. In Fig. 10c, for n=0.49, the system of equations becomes stable, but n=0.49 still being in the neighbourhood of $n_{h}$, the above scenario is witnessed, but with the amplitude of fluctuation slackening over time eventually settles into an optimum biodensity for prey as well as predators.

**Figure 11:**
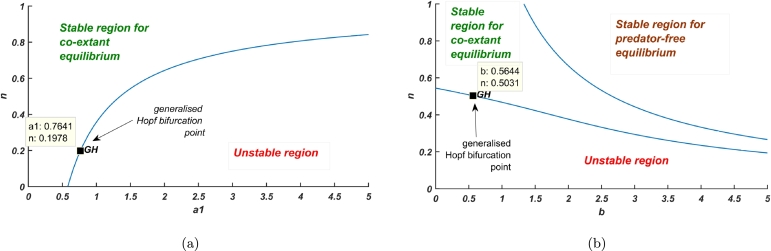
Bifurcation curves in bi-parametric region: (a) predation rate by juveniles *a*_1_ and habitat complexity *n*, and (b) inefficiency rate *b* and habitat complexity *n*.

The portrayal of dynamical scenario with respect to bifurcation in the bi-parametric region is given in Figs. 11(a-b). Hopf bifurcation curve along the parametric axes is drawn thereby dividing the whole region into stable and unstable parts. Fig. 11a shows the relation between predation by juveniles ($a_{1}$) and habitat complexity (*n*). The generalised Hopf bifurcation is at $\left(a_{1},n)=(0.764105,0.197769\right)$, the first Lyapunov coefficient being zero while the second Lyapunov coefficient is −3.700813e−01, the generalised Hopf bifurcation divides the Hopf curve into two parts, supercritical and subcritical parts. Similarly, the Hopf bifurcation curve with respect to the two parameters, inefficiency rate (*b*) and habitat complexity depict the stable and unstable region (Fig. 11b), (b,n)=(0.564633,0.503071) is the generalised Hopf bifurcation point, partitions the Hopf curve into supercritical and subcritical parts. The stable region segregated by Hopf bifurcation curve is further divided into two regions of stability, each for axial and co-extant equilibrium points by transcritical bifurcation curve.

**Figure 12:**
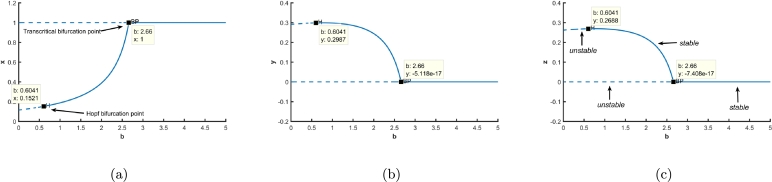
Equilibrium curves with respect to inefficiency rate are depicted for (a) prey population, (b) juvenile predator population and, (c) adult predator population.

The equilibrium curve with respect to the inefficiency rate is visualized in Fig. 12. The Hopf bifurcation point of b is at 0.6040816, for b>0.6040816, the biosystem possess a stable co-extant equilibrium point otherwise unstable, ultimately going to vanishing equilibrium point; which implies the juveniles becoming highly efficient is detrimental to the biosystem. Also on the contrary, them becoming highly inefficient, would cause the whole predator species to be annihilated as the biosystem has a transcritical bifurcation at b=2.66. In Figs. 12(a-c), the solid blue lines depict the stable equilibriums, while the dotted lines depict unstable equilibriums.

**Figure 13:**
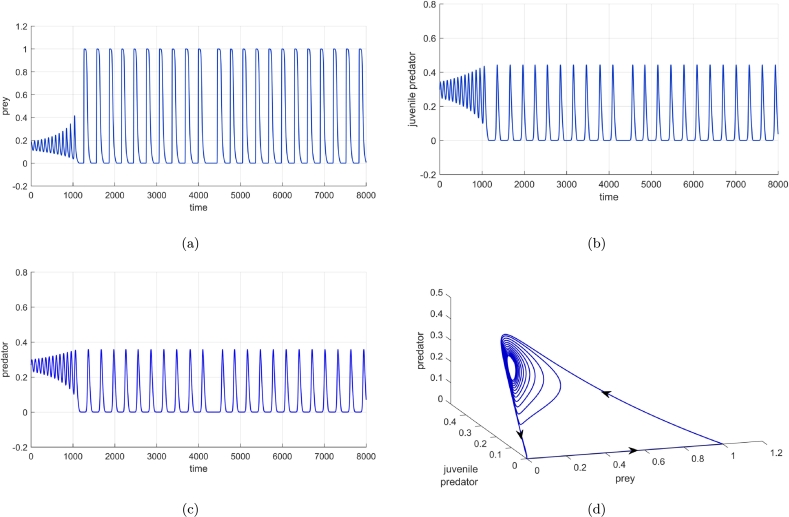
Time series for (a) prey population, (b) juvenile predator population and, (c) adult predator population and, also (d) phase portrait depicting stable limit cycle involving extinction of all (0,0,0), extinction of only predators (1,0,0), and also the co-existence of prey, juvenile predator and matured predators.

For b=0.55, the initial co-existing population is seen to follow a particular trajectory where a cycle spiralling around the co-extant equilibrium point $E_{3}(0.14803,0.29835,0.268515)$ is created between all the three different kinds of equilibrium achievable in our biosystem; from periodic co-existing point $E_{3}^{\prime }(x^{\prime },y^{\prime },z^{\prime })$ to vanishing equilibrium $E_{1}(0,0,0)$ and then to predator-free equilibrium $E_{2}(1,0,0)$, then again to $E_{3}^{\prime }(x^{\prime },y^{\prime },z^{\prime })$. It is worth mentioning that for this particular set of parametric values, neither the conditions of stability of predator-free equilibrium $E_{2}(1,0,0)$ [i.e. $\left(d_{2}(bn+c+d_{1})/c=0.62778)<(a_{3}=1.8\right)$] nor the stability of co-extant equilibrium $E_{3}(0.14803,0.29835,0.268515)$ [i.e. $\xi_{1}=0.562202,\xi_{3}=0.00337,\xi_{4}=-0.00075$] is satisfied. Figs. 13(a-d) show all the three populations fluctuating between the three equilibrium scenarios. Biologically, for some specific parametric values of the inefficiency of juvenile predators, the ecological system can not conform to one particular state of being. When the predator species are at their full potential, the juveniles being sufficiently efficient together with the adult predators start the annihilation of the prey thereby their own too, but when the populace of the predators becomes almost negligible, the prey swiftly reviving from extinction advances towards the environment's carrying capacity. Since the juvenile predators are sufficiently efficient, predator species, too, can revitalize with the availability of its prey.

## 8 Discussion and conclusion

In this paper, a bionetwork comprising of two species, the prey and the predator with stage structure in the predator species has been set forth with ratio-dependent functional response. A new perspective of predation by juvenile predators and the circumstances that effectuate the fatalities of only juvenile predators due to their inefficiency in handling prey along with the prey's habitat, has been explored through both theoretical and numerical methodology. The prey's antipredator behaviour is inferred to be its habitat, without which the prey is innocuous. For example, many insect species such as milkweed bugs sequester phytochemicals as a defensive mechanism against their predators, but the protection gained relies profoundly on the plant species [48]. In the context of spiders, their webs rather than themselves are pernicious to the predators. During predatism, juvenile predators may be inefficient to deal with this habitat complexity, ensuing their death. As is evidenced in the context of *S. nanjingensis* and *T. bambusae*, spiders & snakes, and the others (please refer to introduction for the detailed deliberation). The habitat complexity may also be taken as a territorial intermediate predator, who is at the same stage in the food chain as the juveniles of the top predator, if not higher; in such a scenario barring the territory of the intermediate predator, the juveniles are at liberty to hunt without any repercussions. The intermediate predators are envisaged only as the functioning of the habitat complexity and not as a species in the biosystem.

The biosystem (2) constructed, is proven to dwell in a bounded region in the first octant, hence none of the populations can increase rampantly, which is in conformity with nature's state of affairs. The system of equations manifests three types of equilibrium state. One is obviously the state, where all the populations are extinct, which is an undesirable equilibrium state. The stability is discussed analytically in detail in Appendix C. Biologically, the obtained results imply that if both or either one of the stages of the predator is extinct while the prey persists, the equilibrium state is unachievable as the extinction of one stage would lead to another, while the prey population would follow the logistic growth rate. However, this equilibrium state is reachable if the prey species is extinct along with or without either of the predator stage. The other two manifested equilibrium states are the predator-free equilibrium and the co-extant equilibrium. With the intention of assimilating the biosystem's behaviour in the vicinity of both the equilibrium points, local as well as global stability have been investigated and then visualized graphically; whereupon the acquired results could be implemented for desirable biological scenario.

It is pertinent to note that the co-extant equilibrium point would not exist if the predator-free equilibrium point is stable and also the other way around, as the condition of stability of predator-free equilibrium is $a_{3}c<d_{2}(d_{1}+c+bn)$, while at the same time $a_{3}c>d_{2}(d_{1}+c+bn)$ along with other some other conditions (Theorem 5) are prerequisites for the existence of co-extant equilibrium. Biologically, the predator species would not be able to subsist if either or both of the conversation rate of juvenile ($a_{3}$) and the transition rate from juvenile to adult stage (*c*) is intolerably low. The death rate of adult predators, $d_{2}$ being disproportionately elevated, too, makes the survivability of the predator species non-viable; the same can be said in the context of death rate of juvenile predator ($d_{1}$) and inefficiency rate of juveniles (*b*) together with the rate of habitat complexity (*n*). Needless to say, in such a situation $E_{2}(1,0,0)$ would be stable. Therefore, none of the above circumstances should befall if the existence of predators is to be eventuated. After ensuring co-extant equilibrium point's existence, the perusal of its local stability is done with the help of Routh-Hurwitz criteria. Results concomitant to the perseverance of the biosystem have been looked into. The biosystem persevering implies the system's trajectory is not touching any of the axes, i.e., none of the species would be extinct. For this, the predator-free equilibrium point should be unstable [$a_{3}c>d_{2}(d_{1}+c+bn)$], that is to say the predator would exist, but at the same time the predator species should not overwhelm the prey species, ensuing eradication of both. For that, the obtained result for perseverance has also included $a_{1}(1-n)+a_{2}<m$, by way of explanation, the total predation by both the stages of predators should not exceed their search and capture rate.

It is observed that the predator-free equilibrium point $E_{2}(1,0,0)$ doesn't possess a Hopf bifurcation point. As absenteeism of predators gives carte blanche to the prey whereby they attain their carrying capacity. However transcritical bifurcation does inhabit this equilibrium, the stipulation for which is $a_{3}c=d_{2}(d_{1}+c+bn)$. The transcritical bifurcation occurs on this plane, which serves as a boundary plane, at one side of whose only the predators are extinct and, on the other side, both predator and prey species subsist.

An ample amount of work is done on co-extant equilibrium point due to its ecological importance. Different bifurcation graphs around the co-extant equilibrium $E_{3}$ for various parameters are shown.

Predation by juveniles creates a supplementary slaughter of the prey's population. An increase in $a_{1}$ (predation rate by juvenile) after a critical point is reciprocated by the equilibrium point $E_{3}$ by becoming unstable through Hopf bifurcation, which is observed to be supercritical. Juveniles predating ensure their nutriment while also making them highly efficient for their later stage of life. The more efficient juvenile predators are, the more their survivability would be into the matured stage. Henceforth, decreased inefficiency rate (*b*) after a critical value would indicate an increase in the biomass density of both juveniles and the matured predators initially, correspondingly the biomass of prey would rapidly decrease, which would automatically after a certain period of time result into extinction of all, on the flip side, a great amount of inefficiency would correspond to predator getting completely annihilated and the prey reaching its carrying capacity. Habitat complexity (*n*) appertains to continuity of both the prey and predator species. Decreased complexity of the habitat conforms much to the previous scenario of more predators surviving in their juvenile stage, along with increased deaths of the prey in the hands of the juveniles, whereby leading to the complete extermination of the prey. If either the juvenile predator becomes as efficient as the matured predator (i.e. b=0) or in the absence of habitat complexity (i.e. n=0), the constraints (their inefficiency in dealing with habitat complexity) effectuating the death of juveniles are eliminated, and hence the term implementing the negative repercussions of juveniles hunting vanishes, resulting in both co-extant equilibrium as well axial equilibrium point to be unstable. That being the case, inefficiency rate, as well as habitat complexity, is essential for the survivability of the species. An increase in rate of habitat complexity is allied with the increase in the biomass density of the prey species, and on the flip side decreases the biomass of juvenile and matured predators (Fig. 1). Owing to inefficiency, many migratory bird species incur high mortality of juveniles during their first migration, this dilemma could be curtailed by releasing captive-bred birds at an older age in the subsequent season. Attributable to the dwindling population size of the Egyptian Vulture, this reinforcement has become a necessity to avoid their extinction [46]. On the other hand, the juveniles becoming accustomed to human interference could have either a positive or negative ramification during their handling of a prey with an anti-predator behaviour [41]. On that account, a really controlled environment is required during this whole procedure.

It requires special mention that for some distinctive values of *b*, the biosystem becomes fickle, the vanishing equilibrium, predator-free equilibrium along with the periodic co-existing point all come into play one after another thereby creating a unique stable cycle between them (Fig. 13). From the biological point of view, this can be interpreted as - for low inefficiency rate, the total biomass densities of all three populations decrease and gradually move towards the collapse of the whole biosystem, but as soon as the predators of both stages approach extinction, the prey, who themselves were nearing extinction quickly revives and reaches the carrying capacity of the environment, the almost extinct predators now having the availability of plenty of food, too, revives rapidly. Once all the three populations attain their full potential while co-existing, again start decreasing and going towards the extinction of all, thereby creating this particular cycle where all three kinds of equilibrium can be achieved one after another.

Existence of predators largely relies on the conversion rate of juvenile predator ($a_{3}$), which refers to the amount of nutrients required by the matured predators for reproduction. Diminishing conversion rate insinuates the equilibrium point going towards the predator-free equilibrium point due to the presence of transcritical bifurcation point. Also, an excessive conversion rate culminates extinction for all the three species, as with the increase of juvenile predators, more prey would be killed as a consequence but the prey is not able to further increase their number, hence the extinction. The search and conquer rate of both stages of predators (*m*) is observed to have the ability to destabilize the biosystem for a specific value of *m*. As *m* decreases, the biomass density of the predator species would initially increase due to them being able to kill prey faster and then decrease due to the unavailability of prey. As is evidenced in [39], predation success is extensively impelled by the interaction between encounter and capture probabilities. The hunting behaviour of puma is documented to be determined by the vicuna's trade-off between food and safety, ensuing in uneven distribution of prey availability.

On that account, we can assert that rate of predation by the juvenile ($a_{1}$), their inefficiency (*b*), habitat complexity (*n*), search and conquer rate (*m*) and the conversion rate ($a_{3}$) contributes remarkably in changing the dynamics of our proposed biosystem. All the parameters should be in fair amount for all the species to co-exist.

Some other possible scope of application of our biosystem could be the scenario of date palm trees and its pests such as lesser date moth. Lesser date moth, *Batrachedra amydraula* Meyrick, is grievously baneful for date palm trees, a crop with an enormous economical importance in the Middle-Eastern countries; resulting up to 70-80 percent of yield loss. Natural enemies of the pests, *Goniozus omanensis*, used to exterminate the pests, attack only the larval stage of lesser date moth [49], [50]. Equivalently, in COVID-19 scenario, one may descry the prey population (human being) taking the aid of habitat (places with a low number of infected people along with sanitizer and the likes) to successfully thwart the initial stage of virus (predator) [51]. Here, the rate of predation by the initial stage of predator ($a_{1}$) is taken to be zero.

## Declarations

### Author contribution statement

Debasish Bhattacharjee, Tapasvini Roy, Santanu Acharjee, Tarini Kumar Dutta: conceived and designed the experiments; analyzed and interpreted the data; contributed reagents, materials, analysis tools or data.

Tapasvini Roy: Performed the experiments.

Debasish Bhattacharjee, Tapasvini Roy, Santanu Acharjee: wrote the paper.

### Funding statement

This research did not receive any specific grant from funding agencies in the public, commercial, or not-for-profit sectors.

### Data availability statement

No data was used for the research described in the article.

### Declaration of interests statement

The authors declare no conflict of interest.

### Additional information

No additional information is available for this paper.

## Appendix A Proof of non-negativity

The initial conditions of the biosystem (2) i.e. $x_{0}$, $y_{0}$, $z_{0}$ are all positive, therefore at any time t, we would acquire

$$x(t)=x_{0}\exp[\int_{0}^{t}((1-x(s))-\frac{a_{1}(1-n)y(s)}{m(y(s)+z(s))+(1-n)x(s)}-\frac{a_{2}z(s)}{m(y(s)+z(s))+x(s)})\;ds]\geq 0,y(t)=y_{0}\exp[\int_{0}^{t}(\frac{a_{3}x(s)z(s)}{y(s)(m(y(s)+z(s))+x(s))}-\frac{bnx(s)}{m(y(s)+z(s))+x(s)}-c-d_{1})\;ds]\geq 0,z(t)=z_{0}\exp[\int_{0}^{t}(\frac{cy(s)}{z(s)}-d_{2})\;ds]\geq 0.$$

Hence, the non-negative solution exists.

## Appendix B Proof of boundedness

Defining *p* as $p=a_{3}x+a_{2}y+a_{2}z$, taking the derivative of *p* with respect to time *t*, we have the following equation:

$$\frac{dp}{dt}=a_{3}\frac{dx}{dt}+a_{2}\frac{dy}{dt}+a_{2}\frac{dz}{dt}$$

Now, putting the values of derivatives of *x*, *y*, *z*, we have

$$\frac{dp}{dt}=a_{3}x(1-x)-\frac{a_{1}a_{3}(1-n)xy}{\left(m(y+z)+(1-n)x\right)}-\frac{a_{2}bnxy}{\left(m(y+z)+x\right)}-a_{2}d_{1}y-a_{2}d_{2}z,\text{i.e. }\frac{dp}{dt}\leq a_{3}(1-x)x-d_{1}a_{2}y-d_{2}a_{2}z,$$

$$\text{i.e. }\frac{dp}{dt}+d_{3}p\leq a_{3}(1-x)x+d_{3}a_{3}x-a_{2}y(d_{1}-d_{3})-a_{2}z(d_{2}-d_{3}),\;[\text{where}\;d_{3}=min(d_{2},d_{1})],\text{i.e. }\frac{dp}{dt}+d_{3}p\leq a_{3}x(1+d_{3}-x)=a_{3}(-x^{2}+(1+d_{3})x)=-a_{3}{\left(x-\frac{1+d_{3}}{2}\right)}^{2}+\frac{a_{3}{\left(1+d_{3}\right)}^{2}}{4},\text{i.e. }\frac{dp}{dt}+d_{3}p\leq \frac{a_{3}{\left(1+d_{3}\right)}^{2}}{4}=H\;(\text{say}).$$

Therefore, we have $p\leq \frac{H}{d_{3}}(1-\exp(-d_{3}t))+p(x_{0},y_{0},z_{0})\exp(-d_{3}t)$. When t→∞, we have $p\leq \frac{H}{d_{3}}$. Hence, all solutions of the system (2) are bounded in the region $\left\{(x,y,z)\in {R^{3}}_{+}:p=\frac{H}{d_{3}}-\tau ,\;\text{for any }\tau >0\right\}$.

## Appendix C Stability of vanishing equilibrium point

The Jacobian matrix being undefined at (0,0,0), the usual method for inspecting the stability cannot be done. Hence, the method developed by Arino et al. [33] is used. The biosystem (2)

$$\overset{˙}{X}=G(X)\text{ where }X=\left(\begin{array}{c} x\\ y\\ z \end{array}\right)\text{ and}\;G(X)=\left(\begin{array}{c} x(1-x)-\frac{a_{1}(1-n)xy}{m(y+z)+(1-n)x}-\frac{a_{2}xz}{m(y+z)+x}\\ \frac{a_{3}xz}{m(y+z)+x}-\frac{bnxy}{m(y+z)+x}-cy-d_{1}y\\ cy-d_{2}z \end{array}\right)$$

is transformed to

(6) $$\frac{dX}{dt}=L(X(t))+K(X(t)).$$

Here, L(.) is a homogeneous function of degree 1 which is continuous everywhere except the origin; *K* is a $C^{1}$ function with K(V)=o(V) in the vicinity of (0,0,0),

$$L=\left(\begin{array}{c} x-\frac{a_{1}(1-n)xy}{m(y+z)+(1-n)x}-\frac{a_{2}xz}{m(y+z)+x}\\ \frac{a_{3}xz}{m(y+z)+x}-\frac{bnxy}{m(y+z)+x}-cy-d_{1}y\\ cy-d_{2}z \end{array}\right),\;\text{and}\;K=\left(\begin{array}{c} -x^{2}\\ 0\\ 0 \end{array}\right).$$

If $X_{1}(t)$ is the bounded solution of (6) such that $lim\;inf\;||X_{1}(t)||=0$ and $X_{1}(t_{n},+,.)$ is the subsequence converging to zero, then we define a sequence $\left\{x_{n}\right\}$ by $x_{n}=\frac{X_{1}(t_{n}+s)}{\left||X_{1}(t_{n}+s)|\right|}$ with $||x_{n}||=1$, where ||.|| is the Euclidean norm. A sub-sequence of $\left\{x_{n}\right\}$ is found to be convergent to a function w(t) that satisfies

$$\frac{dw}{dt}=L(w(t))-(w(t),L(w(t)))w(t),$$

with ||w(t)||=1, with the aid of Ascoli-Arzela theorem.

The vector $v(t)=(v_{1},v_{2},v_{3})$ assuages the steady state of *L* by L(v)=(v,L(v))v, which is rewritten as L(v)=ηv.

Now, we are at the stage where we can determine the conditions for reaching the origin with the help of the following:

$$v_{1}(a_{1}(-1+n)v_{2}(v_{1}+m(v_{2}+v_{3}))-a_{2}v_{3}(v_{1}-nv_{1}+m(v_{2}+v_{3}))\;+(v_{1}+m(v_{2}+v_{3}))(v_{1}-nv_{1}+m(v_{2}+v_{3}))\;-(v_{1}+m(v_{2}+v_{3}))(v_{1}-nv_{1}+m(v_{2}+v_{3}))\eta )=0\;a_{3}v_{1}v_{3}-bv_{1}v_{2}-v_{2}(c+d_{1}+\eta )(m(v_{2}+v_{3})+v_{1})=0\;cv_{2}-v_{3}(d_{2}+\eta )=0.$$

For which, we arrive at the following cases:

**Case 1.** Along x−axis i.e. $v_{1}\neq 0,v_{2}=v_{3}=0$:

We have $(\eta -1)(n-1)v_{1}^{3}=0$. Thus, η=1. Hence, (0,0,0) can't be reached along x−axis.

**Case 2.** Along y−axis i.e. $v_{1}=v_{3}=0,v_{2}\neq 0$:

We have $-mv_{2}^{2}(c+d_{1}+\eta )=0$. For $\eta =-(c+d_{1})$, origin is reachable along y−axis.

**Case 3.** Along z−axis i.e. $v_{1}=v_{2}=0,v_{3}\neq 0$:

We have $(\eta +d_{2})v_{3}=0$. For $\eta =-d_{2}$, origin is reachable along z−axis.

**Case 4.** Along xy−plane i.e. $v_{3}=0,v_{1}\neq 0,v_{2}\neq 0$:

There is no possibility of reaching along this plane.

**Case 5.** Along zx−plane i.e. $v_{2}=0,v_{1}\neq 0,v_{3}\neq 0$:

The eigen-value along this plane is η=1, hence unstable.

**Case 6.** Along yz−plane i.e. $v_{1}=0,v_{3}\neq 0,v_{2}\neq 0$:

In the direction of (0,−z,z), the eigen-value is $-c-d_{2}$.

In the direction of $\left(0,y^{\prime },z\right)$, the eigen-value is $-c-d_{1}$, hence stable for both directions, where $y^{\prime }=\frac{-cz-d_{1}z+d_{2}z}{c}$.

## Appendix D Proof of local stability of $E_{1}(1,0,0)$

(7) $$\left(\begin{array}{ccc} -1\; & -a_{1}\; & -a_{2}\\ 0\; & -c-d_{1}-bn\; & a_{3}\\ 0\; & c\; & -d_{2} \end{array}\right)$$

is the Jacobian matrix of the biosystem (2) at axial equilibrium point $E_{2}(1,0,0)$, whose eigen-values are:

−1,

$\frac{1}{2}(-\sqrt{{\left(bn+c+d_{1}+d_{2}\right)}^{2}-4(-a_{3}c+bd_{2}n+cd_{2}+d_{1}d_{2})}-bn-c-d_{1}-d_{2})$, and

$\frac{1}{2}(\sqrt{{\left(bn+c+d_{1}+d_{2}\right)}^{2}-4(-a_{3}c+bd_{2}n+cd_{2}+d_{1}d_{2})}-bn-c-d_{1}-d_{2})$.

The equilibrium point will be stable if all the eigen-values are less than 0. The first two eigen-values are negative. For the third one to be as well, we need

$$a_{3}<\frac{\left(cd_{2}+d_{1}d_{2}+bd_{2}n\right)}{c}.$$

Hence, our proof is done.

## Appendix E Proof of global stability of (1,0,0)

To investigate the globally asymptotically stability of $\left(x_{1},y_{1},z_{1})=(1,0,0\right)$, the following positive definite Lyapunov function is considered.

$$V(x,y,z)=L_{1}(x-x_{1}-x_{1}ln\frac{x}{x_{1}})+L_{2}y+L_{3}z$$

where $L_{1}$, $L_{2}$, $L_{3}$ are positive constants, that are to be chosen appropriately.

Now, taking the time derivative of V(x,y,z) along the solution of the biosystem (2), we have

$$\frac{dV(x,y,z)}{dt}=L_{1}\frac{\left(x-x_{1}\right)}{x}\frac{dx}{dt}+L_{2}\frac{dy}{dt}+L_{3}\frac{dz}{dt},=L_{1}\frac{\left(x-x_{1}\right)}{x}(x(1-x)-\frac{a_{1}(1-n)xy}{m(y+z)+(1-n)x}-\frac{a_{2}xz}{m(y+z)+x})+L_{2}(\frac{a_{3}xz}{m(y+z)+x}-\frac{bnxy}{m(y+z)+x}-cy-d_{1}y)+L_{3}(cy-d_{2}z).$$

Suppose $L_{2}=L_{3}$, and putting $x_{1}=1$, we have,

$$\frac{dV(x,y,z)}{dt}=L_{1}(x-1)((1-x)-\frac{a_{1}(1-n)y}{m(y+z)+(1-n)x}-\frac{a_{2}z}{m(y+z)+x})+L_{2}(\frac{a_{3}xz}{m(y+z)+x}-\frac{bnxy}{m(y+z)+x}-cy-d_{1}y)+L_{2}(cy-d_{2}z)=-L_{1}{\left(x-1\right)}^{2}+\frac{L_{1}(1-x)a_{1}(1-n)y}{m(y+z)+(1-n)x}+\frac{L_{1}a_{2}(1-x)z}{m(y+z)+x})+L_{2}[\frac{a_{3}xz}{m(y+z)+x}-\frac{bnxy}{m(y+z)+x}-d_{1}y-d_{2}z]\leq \frac{L_{1}a_{1}(1-n)(1-x)y}{m(y+z)+(1-n)x}-L_{2}d_{1}y-L_{2}d_{2}z+\frac{L_{1}a_{2}(1-x)z}{m(y+z)+x}+\frac{L_{2}a_{3}xz}{m(y+z)+x}-\frac{L_{2}bnxy}{m(y+z)+x}.$$

Now, putting $L_{1}a_{2}=L_{2}a_{3}$, we have,

$$\frac{dV}{dt}\leq L_{2}[\frac{a_{1}a_{3}(1-n)(1-x)y}{a_{2}(m(y+z)+x(1-n))}-d_{1}y-d_{2}z+\frac{a_{3}z}{m(y+z)+x}-\frac{bnxy}{m(y+z)+x}]\Rightarrow \frac{dV}{dt}<0\;\text{if}\;\frac{a_{1}a_{3}(1-n)(1-x)y}{a_{2}my}<d_{1}y+d_{2}z\;\text{i.e.}\;a_{1}a_{3}(1-n)(1-x)<a_{2}m(d_{1}y+d_{2}z),\text{and}\;\frac{a_{3}z-bnxy}{m(y+z)+x}<0\;\text{i.e.}\;a_{3}z<bnxy.$$

Hence, we obtain the proof.

## Appendix F Proof of local stability of co-extant equilibrium

The Jacobian matrix of our biosystem is given by

(8) $$J(x,y,z)=\left(\begin{array}{ccc} E_{11}\; & E_{12}\; & E_{13}\\ E_{21}\; & E_{22}\; & E_{23}\\ 0\; & c\; & -d_{2} \end{array}\right)$$

Hence,

$$E_{11}=\frac{a_{1}{\left(1-n\right)}^{2}xy}{{\left(m(y+z)+(1-n)x\right)}^{2}}-\frac{a_{1}(1-n)y}{m(y+z)+(1-n)x}-\frac{a_{2}z}{m(y+z)+x}+\frac{a_{2}xz}{{\left(m(y+z)+x\right)}^{2}}-2x+1,E_{12}=-\frac{a_{1}(1-n)x}{m(y+z)+(1-n)x}+\frac{a_{1}m(1-n)xy}{{\left(m(y+z)+(1-n)x\right)}^{2}}+\frac{a_{2}mxz}{{\left(m(y+z)+x\right)}^{2}},E_{13}=\frac{a_{1}m(1-n)xy}{{\left(m(y+z)+(1-n)x\right)}^{2}}-\frac{a_{2}x}{m(y+z)+x}+\frac{a_{2}mxz}{{\left(m(y+z)+x\right)}^{2}},E_{21}=\frac{a_{3}z}{m(y+z)+x}-\frac{a_{3}xz}{{\left(m(y+z)+x\right)}^{2}}-\frac{bny}{m(y+z)+x}+\frac{bnxy}{{\left(m(y+z)+x\right)}^{2}},E_{22}=-\frac{a_{3}mxz}{{\left(m(y+z)+x\right)}^{2}}-\frac{bnx}{m(y+z)+x}+\frac{bmnxy}{{\left(m(y+z)+x\right)}^{2}}-c-d_{1},E_{23}=\frac{a_{3}x}{m(y+z)+x}-\frac{a_{3}mxz}{{\left(m(y+z)+x\right)}^{2}}+\frac{bmnxy}{{\left(m(y+z)+x\right)}^{2}}.$$

Jacobian matrix (8) at the co-extant equilibrium point can be obtained by swapping (x,y,z) with $\left(x^{⁎},y^{⁎},z^{⁎}\right)$, and whose characteristic equation is given below

(9) $$\lambda^{3}+\chi_{1}\lambda^{2}+\chi_{2}\lambda +\chi_{3}=0$$

where,

$$\chi_{1}=-(E_{11}+E_{22}-d_{2}),\chi_{2}=E_{11}E_{22}-E_{22}d_{2}-d_{2}E_{11}-(E_{12}E_{21}+E_{23}c),\chi_{3}=E_{11}(E_{22}d_{2}+E_{23}c)-E_{12}E_{21}d_{2}-E_{13}E_{21}c,\text{and }\chi_{4}=\chi_{1}\chi_{2}-\chi_{3}.$$

By using Routh-Hurwitz criteria, we can see that the point $E_{2}(x^{⁎},y^{⁎},z^{⁎})$ is locally asymptotically stable if $\chi_{1}>0$, $\chi_{3}>0$, and $\chi_{4}>0$.

## Appendix G Proof of global stability of co-extant equilibrium point

The mathematical model can be written as

$$\frac{dX}{dt}=G(X),$$

where,

(10) $$G(X)=\left(\begin{array}{c} x(1-x)-\frac{a_{1}(1-n)xy}{m(y+z)+(1-n)x}-\frac{a_{2}xz}{m(y+z)+x}\\ \frac{a_{3}xz}{m(y+z)+x}-\frac{bnxy}{m(y+z)+x}-cy-d_{1}y\\ cy-d_{2}z \end{array}\right),$$

(11) $$\text{and }\;X=\left(\begin{array}{c} x\\ y\\ z \end{array}\right).$$

For the Jacobian J as given in (8), its second additive compound matrix $J^{\left|2\right|}$, can be written as

$$J^{\left|2\right|}=\left(\begin{array}{ccc} E_{11}+E_{22}\; & E_{23}\; & -E_{13}\\ c\; & E_{11}-d_{2}\; & E_{12}\\ 0\; & E_{21}\; & E_{22}-d_{2} \end{array}\right).$$

Now, let us consider a function M(X) in $C^{1}(D)$ in such a way that $M=\left(\begin{array}{ccc} z/x\; & 0\; & 0\\ 0\; & z/x\; & 0\\ 0\; & 0\; & z/x \end{array}\right)$.

Again, we define

$$M_{G}=\frac{dM}{dX}=\left(\begin{array}{ccc} z/x-\overset{˙}{x}z/x^{2}\; & 0\; & 0\\ 0\; & z/x-\overset{˙}{x}z/x^{2}\; & 0\\ 0\; & 0\; & z/x-\overset{˙}{x}z/x^{2} \end{array}\right).$$

$$∴M_{G}M^{-1}=diag\{\frac{\overset{˙}{z}}{z}-\frac{\overset{˙}{x}}{x},\frac{\overset{˙}{z}}{z}-\frac{\overset{˙}{x}}{x},\frac{\overset{˙}{z}}{z}-\frac{\overset{˙}{x}}{x}\}.$$

$$\text{So, }MJ^{\left|2\right|}M^{-1}=J^{\left|2\right|}.$$

We have,

$$N=M_{G}M^{-1}+MJ^{\left|2\right|}M^{-1}=(\frac{\overset{˙}{z}}{z}-\frac{\overset{˙}{x}}{x})I+J^{\left|2\right|}=\left(\begin{array}{cc} N_{11}\; & N_{12}\\ N_{21}\; & N_{22} \end{array}\right)$$

where,

$$I=\text{identity matrix order 3},N_{11}=\frac{dz/dt}{z}-\frac{dx/dt}{x}+E_{11}+E_{22},N_{12}=\left(\begin{array}{cc} E_{23}\; & -E_{13} \end{array}\right),N_{21}=\left(\begin{array}{c} c\\ 0 \end{array}\right),N_{22}=\left(\begin{array}{cc} E_{11}-d_{2}+\frac{\overset{˙}{z}}{z}-\frac{\overset{˙}{x}}{x}\; & E_{12}\\ E_{21}\; & \frac{\overset{˙}{z}}{z}-\frac{\overset{˙}{x}}{x}+E_{22}-d_{2} \end{array}\right).$$

Considering a vector *(i, j, k)* in $R^{3}$ whose vector norm can be written as |(i,j,k)|=max⁡{|i|,|j|+|k|} and Lozinskii measure with respect to the norm is denoted by Γ.

So, $\Gamma (N)\leq sup\{\xi_{1},\xi_{2}\}$, where $\xi_{1}=\Gamma_{1}(N_{11})+|N_{12}|$, and $\xi_{2}=\Gamma_{1}(N_{22})+|N_{21}|$.

Here $\left|N_{12}\right|$, $\left|N_{21}\right|$ are the matrix norms with respect to the $L^{1}$ vector norm, and $\Gamma_{1}$ is the Lozinskii measure [12], [36] with respect to that norm. Then, the required values can be obtained as

$$\Gamma_{1}(N_{11})=\frac{dz/dt}{z}-\frac{dx/dt}{x}+E_{11}+E_{22},|N_{12}|=Q_{1}=max\{E_{23},|-E_{13}|\},|N_{21}|=max\{c,0\}=c,\text{ and}\;\Gamma_{1}(N_{22})=\frac{\overset{˙}{z}}{z}-\frac{\overset{˙}{x}}{x}+max\{E_{11}+E_{21}-d_{2},E_{22}+E_{12}-d_{2}\}=\frac{dz/dt}{z}-\frac{dx/dt}{x}+Q_{2},\text{where}\;Q_{2}=max\{E_{11}+E_{21}-d_{2},E_{22}+E_{12}-d_{2}\}.$$

Again, from the first equation of the biosystem (2)

$$\frac{dx/dt}{x}=1-x-\frac{a_{1}(1-n)y}{m(y+z)+x(1-n)}-\frac{a_{2}z}{m(y+z)+x}.$$

Now,

$$\xi_{1}=\Gamma_{1}(N_{11})+|N_{12}|=\frac{dz/dt}{z}-\frac{dx/dt}{x}+E_{11}+E_{22}+Q_{1},\text{and }\xi_{2}=\Gamma_{1}(N_{22})+|N_{21}|=\frac{dz/dt}{z}-\frac{dx/dt}{x}+Q_{2}+c.$$

Next,

$$\Gamma (N)\leq max\{\xi_{1},\xi_{2}\}$$

$$\text{i.e. }\Gamma (N)\leq \frac{dz/dt}{z}-\frac{dx/dt}{x}+max\{E_{11}+E_{22}+Q_{1},Q_{2}+c\},\text{i.e. }\Gamma (N)\leq \frac{dz/dt}{z}-\frac{dx/dt}{x}+Q,\text{where }Q=max\{E_{11}+E_{22}+Q_{1},Q_{2}+c\}.$$

$$\text{Thus, }\Gamma (N)\leq \frac{\overset{˙}{z}}{z}+\vartheta ,\text{ where }\vartheta =Q-\frac{\overset{˙}{x}}{x}.$$

We consider *α* which is a positive real number and T≥0, such that α=inf{x(t),y(t),z(t)} when t>T.

Also, we would have

$$Q_{1}=max\{|E_{23}|,|-E_{13}|\}.∴Q_{1}=max\{|\frac{a_{3}}{2m+1}-\frac{a_{3}m}{{\left(2m+1\right)}^{2}}+\frac{bmn}{{\left(2m+1\right)}^{2}}|,|-\frac{a_{1}m(1-n)}{{\left(m2+(1-n)\right)}^{2}}+\frac{a_{2}}{m2+1}-\frac{a_{2}m}{{\left(m2+1\right)}^{2}}|\}\text{at }\;\alpha =inf\{x(t),y(t),z(t)\}.$$

Similarly,

$$Q_{2}=max\{E_{11}+E_{21}-d_{2},E_{22}+E_{12}-d_{2}\}=max\{\frac{a_{1}{\left(1-n\right)}^{2}}{{\left(m2+(1-n)\right)}^{2}}-\frac{a_{1}(1-n)}{m2+(1-n)}-\frac{a_{2}}{m2+1}+\frac{a_{2}}{{\left(m2+1\right)}^{2}}-2\alpha +1+\frac{a_{3}}{2m+1}-\frac{a_{3}}{{\left(2m+1\right)}^{2}}-\frac{bn}{2m+1}+\frac{bn}{{\left(2m+1\right)}^{2}}-d_{2},-\frac{a_{3}m}{{\left(2m+1\right)}^{2}}-\frac{bn}{2m+1}+\frac{bmn}{{\left(2m+1\right)}^{2}}-c-d_{1}-\frac{a_{1}(1-n)}{m2+(1-n)}+\frac{a_{1}m(1-n)}{{\left(m2+(1-n)\right)}^{2}}+\frac{a_{2}m}{{\left(m2+1\right)}^{2}}-d_{2}\}\;\text{at}\;\alpha =inf\{x(t),y(t),z(t)\}.$$

Also, at *α*,

$$Q=max\{E_{11}+E_{22}+Q_{1},Q_{2}+c\}=max\{\frac{a_{1}{\left(1-n\right)}^{2}}{{\left(m2+(1-n)\right)}^{2}}-\frac{a_{1}(1-n)}{m2+(1-n)}-\frac{a_{2}}{m2+1}+\frac{a_{2}}{{\left(m2+1\right)}^{2}}-2\alpha +1-\frac{a_{3}m}{{\left(2m+1\right)}^{2}}-\frac{bn}{2m+1}+\frac{bmn}{{\left(2m+1\right)}^{2}}-c-d_{1}+Q_{1},Q_{2}+c\},\text{and }\overset{˙}{x}/x=1-\alpha -\frac{a_{1}(1-n)}{2m+1-n}-\frac{a_{2}}{2m+1}.$$

Hence, we would get *ϑ* at *α*. Therefore, we have

$$\Gamma (N)\leq \frac{\overset{˙}{z}}{z}+\vartheta \int_{0}^{t}\Gamma (N)\;ds\leq \int_{0}^{t}\frac{dz/dt}{z}\;dt+\vartheta \int_{0}^{t}1\;dt\;\text{i.e. }\int_{0}^{t}\Gamma (N)\;ds\leq log\frac{z(t)}{z(0)}+\vartheta t\;\text{i.e. }\frac{1}{t}\int_{0}^{t}\Gamma (N)\;ds\leq \frac{1}{t}log\frac{z(t)}{z(0)}+\vartheta \;\lim_{t\to \infty }\;sup\;\frac{1}{t}\int_{0}^{t}\Gamma (N)\;ds<\vartheta <0.$$

Thus, the system of equations is globally asymptotically stable around its co-extant equilibrium point, when ϑ<0.

## Appendix H Proof of perseverance

At the outset, we shall find the condition for which x(t)>0 for all t>0, from the first equation of our biosystem (2) we have the following:

$$\overset{˙}{x}=x(1-x)-\frac{a_{1}(1-n)xy}{m(y+z)+x(1-n)}-\frac{a_{2}xz}{m(y+z)+x}\geq x(1-x)-\frac{a_{1}(1-n)xy}{my}-\frac{a_{2}xz}{mz}=x(1-x-a_{1}(1-n)/m-a_{2}/m).$$

$$\text{So, }\lim_{t\to \infty }\;inf\;x(t)\geq 1-\frac{a_{1}(1-n)+a_{2}}{m}=L\text{ (say)}$$

Thus, L>0 i.e., $m>a_{1}(1-n)+a_{2}$. If the above condition is satisfied then, for the biosystem the vanishing equilibrium point cannot be achieved as the prey would always exist. The next plausible manifestation would be either the predator free equilibrium point or the co-extant equilibrium point. Now from the previous stability theorem of the axial equilibrium point (3), if we take $a_{3}c>d_{2}(bn+c+d_{1})$, then the predator free equilibrium point too would be unstable. Hence, the only possible scenario left would be the co-existence of all three populations. Moreover without the stability of the co-extant equilibrium point, there would exist some bounded trajectory in the positive octant showing co-existence of the populations. Therefore, we can say, the biosystem (2) perseveres if

$$m>a_{1}(1-n)+a_{2}\text{ and }a_{3}c>d_{2}(bn+c+d_{1}).$$

## Appendix I Criteria of Hopf bifurcation

If $n_{h}$ is assumed to be the point of Hopf bifurcation for the parameter *n*, then the necessary and sufficient condition for Hopf bifurcation at $n_{h}$ is

(i) $\chi_{1},\chi_{2}>0$, $\chi_{3}=\chi_{1}\chi_{2}$. $\chi_{i}$, i=1,2,3 are from (9),
(ii) $\frac{d}{dn}{\left(Re(\lambda_{i}(n))\right)}_{n=n_{h}}\neq 0$ for i=1,2,3.

The characteristic equation given in (9) is

(12) $$\lambda^{3}+\lambda^{2}\chi_{1}+\lambda \chi_{2}+\chi_{3}=0,\text{for}\;\chi_{3}=\chi_{1}\chi_{2}\text{ at }n=n_{h}.\text{So, }\lambda^{3}+\lambda^{2}\chi_{1}+\lambda \chi_{2}+\chi_{1}\chi_{2}=0,\text{i.e. }\lambda^{2}(\lambda +\chi_{1})+\chi_{2}(\lambda +\chi_{1})=0,\text{i.e. }(\lambda^{2}+\chi_{2})(\lambda +\chi_{1})=0.\text{Thus, }\lambda =-\chi_{1},\;\pm \sqrt{\chi_{2}}i\text{ if }\chi_{1},\chi_{2}\text{ are positive}.$$

For $n\in (n_{h}-\epsilon ,n_{h}+\epsilon )$, the general forms of the roots are

$\lambda_{1}=\Psi_{1}(n)+i\Psi_{2}(n)$,

$\lambda_{2}=\Psi_{1}(n)-i\Psi_{2}(n)$, and

$\lambda_{3}=-\chi_{1}(n)$.

Now, for transversality condition, we need to establish

$$\frac{d}{dn}{\left(Re(\lambda_{i}(n))\right)}_{n=n_{h}}\neq 0\;\text{for}\;i=1,2,3.$$

Substituting $\lambda_{1}=\Psi_{1}(n)+i\Psi_{2}(n)$ in (12), we have

(13) $$(\Psi_{1}^{2}-\Psi_{2}^{2}+2i\Psi_{1}\Psi_{2}+\chi_{2})(\Psi_{1}+i\Psi_{2}+\chi_{1})=0.$$

Differentiating (13) and separating the real and imaginary part we get the following:

$$P_{1}(n)\Psi_{1}^{\prime }(n)-P_{2}(n){\Psi^{\prime }}_{2}+R_{1}(n)=0,$$

$$\text{and }P_{2}(n){\Psi^{\prime }}_{1}(n)+P_{1}(n){\Psi^{\prime }}_{2}+R_{2}(n)=0.$$

Here, $P_{1}=3\Psi_{1}^{2}-3\Psi_{2}^{2}+\chi_{2}+2\chi_{1}\Psi_{1}$,

$P_{2}=6\Psi_{1}\Psi_{2}+2\Psi_{2}\chi_{1}$,

$R_{1}=\chi_{1}^{\prime }\Psi_{1}^{2}-\chi_{1}^{\prime }\Psi_{2}^{2}+\chi_{2}^{\prime }\Psi_{1}+\chi_{3}^{\prime }$,

and $R_{2}=\chi_{2}^{\prime }\Psi_{2}+2\chi_{1}^{\prime }\Psi_{1}\Psi_{2}$.

Using $\Psi_{1}(n_{h})=0$ and $\Psi_{2}(n_{h})=i\sqrt{\chi_{2}(n_{h})}$, we get

$$P_{1}(n_{h})=-3\chi_{2}+\chi_{2}=-2\chi_{2},\;P_{2}(n_{h})=2\chi_{1}\sqrt{\chi_{2}},$$

$$\text{and }R_{1}(n_{h})=\chi_{3}^{\prime }-\chi_{1}^{\prime }\chi_{2},\;\text{and }R_{2}(n_{h})=\sqrt{\chi_{2}}\chi_{2}^{\prime }.$$

Hence,

$$\frac{d}{dn}{\left[Re(\lambda_{1,2}(n))\right]}_{n=n_{h}}=\Psi_{1}=-\frac{P_{1}R_{1}+P_{2}R_{2}}{P_{1}^{2}+P_{2}^{2}}=-\frac{-2\chi_{2}(\chi_{3}^{\prime }-\chi_{1}^{\prime }\chi_{2})+2\chi_{1}\chi_{2}\chi_{2}^{\prime }}{4\chi_{2}^{2}+4\chi_{1}^{2}\chi_{2}}=-\frac{\chi_{1}^{\prime }\chi_{2}-\chi_{3}^{\prime }+\chi_{1}\chi_{2}^{\prime }}{2\chi_{2}+2\chi_{1}^{2}}\neq 0,$$

$$\text{if }\chi_{1}^{\prime }\chi_{2}-\chi_{3}^{\prime }+\chi_{1}\chi_{2}^{\prime }\neq 0.$$

$$\text{So, }\;\frac{d}{dn}(\chi_{1}\chi_{2}-\chi_{3})\neq 0\;\text{at}\;n=n_{h}.$$

Therefore, the transversality conditions hold. Hence, the biosystem (2) undergoes Hopf bifurcation around the positive interior equilibrium point $E_{2}(x^{⁎},y^{⁎},z^{⁎})$, when the habitat complexity (*n*) crosses the critical value $n=n_{h}$.

Existence of $n_{h}$ and its direction are shown numerically in section 7.2.

## Appendix J Direction and stability of bifurcating solutions

Here, we endeavour to determine the direction and stability criterion of the bifurcating periodic solution arising from Hopf bifurcation with the help of procedure given by Hassard et al. [37], where the set of differential equations of the system (2) is reduced into its normal form.

We introduce new variables ($x^{\prime },y^{\prime },z^{\prime }$) as $x=x^{\prime }+x^{⁎}$, $y=y^{\prime }+y^{⁎}$, $z=z^{\prime }+z^{⁎}$. For simplicity, $x^{\prime }\to x$, $y^{\prime }\to y$, $z^{\prime }\to z$. The system of equation (2) is transformed to

$$\frac{dx}{dt}=(x+x^{⁎})(1-(x+x^{⁎}))-\frac{a_{2}(x+x^{⁎})(z+z^{⁎})}{m(y+z+y^{⁎}+z^{⁎})+(x+x^{⁎})}-\frac{a_{1}(1-n)(x+x^{⁎})(y+y^{⁎})}{m(y+z+y^{⁎}+z^{⁎})+(x+x^{⁎})(1-n)},\frac{dy}{dt}=\frac{a_{3}(x+x^{⁎})(z+z^{⁎})}{m(y+z+y^{⁎}+z^{⁎})+(x+x^{⁎})}-c(y+y^{⁎})-\frac{bn(x+x^{⁎})(y+y^{⁎})}{m(y+z+y^{⁎}+z^{⁎})+(x+x^{⁎})}-d_{1}(y+y^{⁎}),\text{and }\frac{dz}{dt}=c(y+y^{⁎})-d_{2}(z+z^{⁎}).$$

Partitioning the above equations into linear and nonlinear parts, we get

(14) $$\overset{˙}{x}=ln_{1}+nln_{1},\;\overset{˙}{y}=ln_{2}+nln_{2},\;\text{and }\overset{˙}{z}=ln_{3}+nln_{3}.$$

Here, $ln_{i}$ (i=1,2,3) and $nln_{i}$ (i=1,2,3) are the linear and nonlinear parts respectively. So, (14) is written as given below:

(15) $$\overset{˙}{X}=RX+S,$$

where *R* is the Jacobian matrix of the linear part and *S* is the nonlinear part.

$$X=\left(\begin{array}{c} x\\ y\\ z \end{array}\right),\;R=\left(\begin{array}{ccc} r_{11}\; & r_{12}\; & r_{13}\\ r_{21}\; & r_{22}\; & r_{23}\\ r_{31}\; & r_{32}\; & r_{33} \end{array}\right),\;S=\left(\begin{array}{c} S_{1}\\ S_{2}\\ S_{3} \end{array}\right),$$

where,

$$r_{11}=\left(\frac{a_{1}(n-1)y^{⁎}m(y^{⁎}+z^{⁎})}{{\left(m(y^{⁎}+z^{⁎})-nx^{⁎}+x^{⁎}\right)}^{2}}-\frac{a_{2}z^{⁎}m(y^{⁎}+z^{⁎})}{{\left(m(y^{⁎}+z^{⁎})+x^{⁎}\right)}^{2}}\right)-2x^{⁎}+1,r_{12}=\left(\frac{a_{2}mz^{⁎}x^{⁎}}{{\left(m(y^{⁎}+z^{⁎})+x^{⁎}\right)}^{2}}-\frac{a_{1}x^{⁎}({\left(n-1\right)}^{2}x^{⁎}-(1-n)mz^{⁎})}{{\left(m(y^{⁎}+z^{⁎})-nx^{⁎}+x^{⁎}\right)}^{2}}\right),r_{13}=\left(-\frac{a_{1}m(n-1)y^{⁎}x^{⁎}}{{\left(m(y^{⁎}+z^{⁎})-nx^{⁎}+x^{⁎}\right)}^{2}}-\frac{a_{2}(my^{⁎}+x^{⁎})x^{⁎}}{{\left(m(y^{⁎}+z^{⁎})+x^{⁎}\right)}^{2}}\right),r_{21}=\frac{-mz^{⁎}(bny^{⁎}-a_{3}y^{⁎})+a_{3}mz^{⁎2}-bmny^{⁎2}}{{\left(m(y^{⁎}+z^{⁎})+x^{⁎}\right)}^{2}},r_{22}=[-mz^{⁎}(a_{3}x^{⁎}+bnx^{⁎})-bnx^{⁎2}-c{\left(m(y^{⁎}+z^{⁎})+x^{⁎}\right)}^{2}-d_{1}{\left(m(y^{⁎}+z^{⁎})+x^{⁎}\right)}^{2}]/[{\left(m(y^{⁎}+z^{⁎})+x^{⁎}\right)}^{2}],r_{23}=\frac{a_{3}mx^{⁎}y^{⁎}+a_{3}x^{⁎2}+bmnx^{⁎}y^{⁎}}{{\left(m(y^{⁎}+z^{⁎})+x^{⁎}\right)}^{2}},r_{31}=0,\;r_{32}=c,\;r_{33}=-d_{2}.$$

Now we consider two conjugate imaginary eigen-values $\lambda_{1,2}=\pm \beta$ and other eigen-value $\lambda_{3}=v_{1}$ [where $\beta =i\sqrt{\eta_{2}}$] of the characteristic equation (9).

We procure a transformation matrix T such that

$$T^{-1}RT=\left(\begin{array}{ccc} 0\; & -\beta \; & 0\\ \beta \; & 0\; & 0\\ 0\; & 0\; & v \end{array}\right).$$

Now, T is a non-singular matrix which is given as

$$T=\left(\begin{array}{ccc} 1\; & 0\; & 1\\ t_{21}\; & t_{22}\; & t_{23}\\ t_{31}\; & t_{32}\; & t_{33} \end{array}\right),$$

where,

$$t_{21}=[r_{23}(r_{21}r_{32}r_{33}+r_{22}r_{31}r_{33}-r_{31}\beta^{2})-r_{21}r_{22}(r_{33}^{2}+\beta^{2})-r_{23}^{2}r_{31}r_{32}]/[\left(r_{22}^{2}+\beta^{2}\right)\left(r_{33}^{2}+\beta^{2}\right)+2r_{23}r_{32}\left(\beta^{2}-r_{22}r_{33}\right)+r_{23}^{2}r_{32}^{2}],t_{22}=\frac{\beta \left(r_{21}\left(r_{23}r_{32}+r_{33}^{2}+\beta^{2}\right)-r_{22}r_{23}r_{31}-r_{23}r_{31}r_{33}\right)}{\left(r_{22}^{2}+\beta^{2}\right)\left(r_{33}^{2}+\beta^{2}\right)+2r_{23}r_{32}\left(\beta^{2}-r_{22}r_{33}\right)+r_{23}^{2}r_{32}^{2}},t_{23}=\frac{r_{21}(v-r_{33})+r_{23}r_{31}}{(r_{22}-v)(r_{33}-v)-r_{23}r_{32}},t_{31}=-[-r_{22}r_{32}(r_{21}r_{33}+r_{23}r_{31})+r_{21}r_{32}\left(r_{23}r_{32}+\beta^{2}\right)+r_{22}^{2}r_{31}r_{33}+r_{31}r_{33}\beta^{2}]/[\left(r_{22}^{2}+\beta^{2}\right)\left(r_{33}^{2}+\beta^{2}\right)+2r_{23}r_{32}\left(\beta^{2}-r_{22}r_{33}\right)+r_{23}^{2}r_{32}^{2}],t_{32}=\frac{\beta \left(-r_{21}r_{22}r_{32}-r_{21}r_{32}r_{33}+r_{22}^{2}r_{31}+r_{23}r_{31}r_{32}+r_{31}\beta^{2}\right)}{\left(r_{22}^{2}+\beta^{2}\right)\left(r_{33}^{2}+\beta^{2}\right)+2r_{23}r_{32}\left(\beta^{2}-r_{22}r_{33}\right)+r_{23}^{2}r_{32}^{2}},t_{33}=\frac{r_{21}r_{32}-r_{22}r_{31}+r_{31}v}{(r_{22}-v)(r_{33}-v)-r_{23}r_{32}}.$$

Again, we make another change of variable to obtain the normal form of (15).

We consider X=TY.

$$\text{Hence, }\overset{˙}{Y}=T^{-1}RTY+T^{-1}S,$$

$$\text{i.e.}\;\overset{˙}{Y}=(T^{-1}RT)Y+U,$$

where $U=\left(\begin{array}{c} U_{1}\\ U_{2}\\ U_{3} \end{array}\right)$. Here, $U_{i}(y_{1},y_{2},y_{3})$ i=1,2,3 can be obtained by transforming $S_{i}$'s using the variables $x=y_{1}+y_{3}$, $y=t_{21}y_{1}+t_{22}y_{2}+t_{23}y_{3}$, $z=t_{31}y_{1}+t_{32}y_{2}+t_{33}y_{3}$.

Now we derive the expressions $g_{11}$, $g_{02}$, $g_{20}$, $G_{21}$, $h_{11}^{1}$, $h_{20}^{1}$, $w_{11}$, $w_{20}$, $G_{110}^{1}$, $G_{101}^{1}$, $g_{21}$ at $\left(y_{1},y_{2},y_{3})=(0,0,0\right)$.

$$g_{11}=\frac{1}{4}\left(\frac{\partial^{2}U_{1}}{\partial y_{1}\;\partial y_{1}}+\frac{\partial^{2}U_{1}}{\partial y_{2}\;\partial y_{2}}+i\left(\frac{\partial^{2}U_{2}}{\partial y_{1}\;\partial y_{1}}+\frac{\partial^{2}U_{2}}{\partial y_{2}\;\partial y_{2}}\right)\right),g_{02}=\frac{1}{4}[i\left(2\frac{\partial^{2}U_{1}}{\partial y_{1}\;\partial y_{2}}+\frac{\partial^{2}U_{2}}{\partial y_{1}\;\partial y_{1}}-\frac{\partial^{2}U_{2}}{\partial y_{2}\;\partial y_{2}}\right)+\frac{\partial^{2}U_{1}}{\partial y_{1}\;\partial y_{1}}-\frac{\partial^{2}U_{1}}{\partial y_{2}\;\partial y_{2}}-2\frac{\partial^{2}U_{2}}{\partial y_{1}\;\partial y_{2}}],g_{20}=\frac{1}{4}[i\left(-2\frac{\partial^{2}U_{1}}{\partial y_{1}\;\partial y_{2}}+\frac{\partial^{2}U_{2}}{\partial y_{1}\;\partial y_{1}}-\frac{\partial^{2}U_{2}}{\partial y_{2}\;\partial y_{2}}\right)+\frac{\partial^{2}U_{1}}{\partial y_{1}\;\partial y_{1}}-\frac{\partial^{2}U_{1}}{\partial y_{2}\;\partial y_{2}}+2\frac{\partial^{2}U_{2}}{\partial y_{1}\;\partial y_{2}}],G_{21}=\frac{1}{8}[i(-\frac{\partial^{3}U_{1}}{\partial y_{1}\;\partial y_{1}\;\partial y_{2}}-\frac{\partial^{3}U_{1}}{\partial y_{2}\;\partial y_{2}\;\partial y_{2}}+\frac{\partial^{3}U_{2}}{\partial y_{1}\;\partial y_{2}\;\partial y_{2}}+\frac{\partial^{3}U_{2}}{\partial y_{1}\;\partial y_{1}\;\partial y_{1}})+\frac{\partial^{3}U_{1}}{\partial y_{1}\;\partial y_{2}\;\partial y_{2}}+\frac{\partial^{3}U_{1}}{\partial y_{1}\;\partial y_{1}\;\partial y_{1}}+\frac{\partial^{3}U_{2}}{\partial y_{1}\;\partial y_{1}\;\partial y_{2}}+\frac{\partial^{3}U_{2}}{\partial y_{2}\;\partial y_{2}\;\partial y_{2}}],$$

$$G_{110}^{1}=\frac{1}{2}\left(i\left(\frac{\partial^{2}U_{2}}{\partial y_{1}\;\partial y_{3}}-\frac{\partial^{2}U_{1}}{\partial y_{2}\;\partial y_{3}}\right)+\frac{\partial^{2}U_{1}}{\partial y_{1}\;\partial y_{3}}+\frac{\partial^{2}U_{2}}{\partial y_{2}\;\partial y_{3}}\right),G_{101}^{1}=\frac{1}{2}\left(i\left(\frac{\partial^{2}U_{1}}{\partial y_{2}\;\partial y_{3}}+\frac{\partial^{2}U_{2}}{\partial y_{1}\;\partial y_{3}}\right)+\frac{\partial^{2}U_{1}}{\partial y_{1}\;\partial y_{3}}-\frac{\partial^{2}U_{2}}{\partial y_{2}\;\partial y_{3}}\right),h_{11}^{1}=\frac{1}{4}\left(\frac{\partial^{2}U_{3}}{\partial y_{1}\;\partial y_{1}}+\frac{\partial^{2}U_{3}}{\partial y_{2}\;\partial y_{2}}\right),h_{20}^{1}=\frac{1}{4}\left(-2i\frac{\partial^{2}U_{3}}{\partial y_{1}\;\partial y_{2}}+\frac{\partial^{2}U_{3}}{\partial y_{1}\;\partial y_{1}}-\frac{\partial^{2}U_{3}}{\partial y_{2}\;\partial y_{2}}\right),v_{1}w_{11}=-h_{11}^{1},(v_{1}-2i\beta )w_{20}=-h_{20}^{1},\text{and }g_{21}=G_{21}+2G_{110}^{1}w_{11}+G_{101}^{1}w_{20}.$$

Now, calculating the quantities, we get the following

$$C_{1}(0)=\frac{i}{2\beta }(g_{11}g_{20}-2|g_{11}{|}^{2}-\frac{1}{3}|g_{02}{|}^{2})+\frac{1}{2}g_{21},$$

$$\mu_{2}=-ReC_{1}(0)/\alpha^{\prime }(0),$$

$$T_{2}=-(Im(C_{1}(0))+\mu_{2}\beta^{\prime }(0))/(\beta ),$$

$$\beta_{2}=2ReC_{1}(0),$$

where $\alpha^{\prime }(0)=Re\lambda_{1}^{\prime }(n_{h})$, $\beta^{\prime }(0)=Im\lambda_{1}^{\prime }(n_{h})$. Here, $n_{h}$ is the bifurcation point and $\lambda_{1}(n_{h})=i\beta$.

## Appendix K Proof of transcritical bifurcation

The Jacobian matrix of the biosystem at the equilibrium point $E_{1}(1,0,0)$ is as given in (7). One of its eigen-values will be zero if $a_{3}c=d_{2}(c+d_{1}+bn)$ and other two are negative. Taking *b* as the bifurcating parameter i.e. $b=b_{t}=\frac{a_{3}c-cd_{2}-d_{1}d_{2}}{nd_{2}}$.

For $b=b_{t}$, the variational matrix at $E_{1}$ is given below:

$$J_{1}=\left(\begin{array}{ccc} -1\; & a_{1}\; & -a_{2}\\ 0\; & -\frac{a_{3}c}{d_{2}}\; & a_{3}\\ 0\; & c\; & -d_{2} \end{array}\right).$$

Then, $W_{1}={\left(v_{1},v_{2},v_{3}\right)}^{t}={\left(-\left(\frac{a_{2}c+a_{1}d_{2}}{d_{2}}\right)v_{2},v_{2},\frac{cv_{2}}{d_{2}}\right)}^{t}$ and $W_{2}={\left(w_{1},w_{2},w_{3}\right)}^{t}={\left(0,w_{2},\frac{a_{3}w_{2}}{d_{2}}\right)}^{t}$ are the eigen-vectors corresponding to the zero eigen-value for the matrices $J_{1}$ and $J_{1}^{t}$ respectively. The biosystem (2) is written in the form $\overset{˙}{X}=G(X)$ as is in ((10) and (11)). Applying Sotomayor theorem [38] for transcritical behaviour, we have the following:

$$W_{2}^{t}G_{b}(E_{1};b_{t})=0,\;W_{2}^{t}(DG_{b}(E_{1};b_{t})V)\neq 0$$

and

$$W_{2}^{t}[D^{2}G(E_{1};b_{t})V]\neq 0,$$

$$G_{b}(E_{1};b_{t})={\left(\begin{array}{c} 0\\ -\frac{nxy}{m(y+z)+x}\\ 0 \end{array}\right)}_{\left(at\;(1,0,0),b_{t}\right)}=\left(\begin{array}{c} 0\\ 0\\ 0 \end{array}\right),$$

$$DG_{b}(E_{1};b_{t})=\left(\begin{array}{ccc} 0\; & 0\; & 0\\ 0\; & -n\; & 0\\ 0\; & 0\; & 0 \end{array}\right),\;D^{2}G(E_{1};b_{t})V=\left(\begin{array}{c} \gamma_{1}\\ \gamma_{2}\\ \gamma_{3} \end{array}\right),$$

where

$$\gamma_{1}=v_{2}^{2}\left(-\frac{2{\left(a_{1}d_{2}+a_{2}c\right)}^{2}}{d_{2}^{2}}+\frac{cm\left(\frac{a_{1}{\left(n-1\right)}^{2}}{{\left(1-n\right)}^{3}}+a_{2}\right)}{d_{2}}-\frac{2a_{1}m}{n-1}+\frac{2a_{2}c^{2}m}{d_{2}^{2}}\right),\gamma_{2}=-\frac{mv_{2}^{2}(a_{3}c(c-d_{2})+d_{2}(c+d_{1})(c+2d_{2}))}{d_{2}^{2}},\gamma_{3}=0.$$

Hence,

$$W_{2}^{t}G_{b}(E_{1};b_{t})=0,W_{2}^{t}(DG_{b}(E_{1};b_{t})V)=-nw_{2}v_{2}\neq 0,W_{2}^{t}[D^{2}G(E_{1};b_{t})V]=-\frac{mv_{2}^{2}(a_{3}c(c-d_{2})+d_{2}(c+d_{1})(c+2d_{2}))}{d_{2}^{2}}w_{2}\neq 0.$$

Hence, our result is proven.
